# Market interactions, trust and reciprocity

**DOI:** 10.1371/journal.pone.0232704

**Published:** 2020-05-07

**Authors:** Ginny Seung Choi, Virgil Henry Storr

**Affiliations:** 1 F.A. Hayek Program for Advanced Study in Philosophy, Politics and Economics, Mercatus Center at George Mason University, Fairfax, VA, United States of America; 2 Department of Economics, George Mason University, Fairfax, VA, United States of America; Middlesex University, UNITED KINGDOM

## Abstract

Trust is a key ingredient of almost all market interactions. Much of the literature on the relationship between trust and market activity, however, has focused on how trust facilitates market activity rather than on how market activity affects trust. In this study, however, we investigate whether market interactions can affect the subsequent trusting and reciprocating behavior of former trading partners. Additionally, we explore the effect of personal and impersonal exchange on the trusting and reciprocating behavior of former trading partners. We find experimental evidence that suggests that positive and negative market interactions can affect such behavior. Further, we find that past market dealings only affect the trusting and reciprocating behavior of subjects who participated in an experimental market where exchanges were more personal, but did not affect trust and reciprocity between trading partners who participated in an experimental market where exchanges were more impersonal. In the market where exchanges are more personal, people exhibit higher levels of trust and reciprocity to trading partners with whom they have mostly positive market interactions than with whom they have mostly negative market interactions. However, in the market where exchanges are more impersonal, people exhibit the same levels of trust and reciprocity to trading partners regardless of the nature of their previous market interactions.

## Introduction

Trust is a key ingredient of almost all market interactions [1]. Trust facilitates market exchanges by lowering transactions costs since it would be costly (perhaps prohibitively) to address all possible contingencies of a transaction. As Arrow [2, p. 357] stated, “Virtually every commercial transaction has within itself an element of trust… It can be plausibly argued that much of the economic backwardness in the world can be explained by a lack of mutual confidence.” Trust as a facilitator of market interactions has been widely studied [3–7]. But less attention has been given to whether or not market transactions can facilitate the emergence of trust. Specifically, the potential of markets to allow for the emergence of social relationships characterized by trust and reciprocity has been relatively understudied.

The market, however, is a social space where meaningful social connections can and do develop [8–12]. Certainly, as orthodox (i.e. neoclassical) economics implies, the market is the site where buyers and sellers negotiate and exchange goods, information, and other resources. However, it is also a space where buyers and sellers get together, interact and converse with one another. Because people are not automatons, these market conversations and interactions tend to extend beyond strictly economic topics and terrain. Think, for instance, of the conversations between hairdressers and their clients, the relationships between children and their caretakers, and the connections between colleagues in an office or on the factory floor. These market relationships often turn into deep social connections characterized by trust and reciprocity. While qualitative evidence has established that meaningful social relationships characterized by trust and reciprocity can and do emerge between economic actors [13–18], there have been relatively few quantitative and even fewer experimental studies that have examined the emergence of trusting relationships between market actors and how different market institutions facilitate the formation of such relationships.

In this study, we used a laboratory experiment to study whether positive and negative market interactions can affect the trusting and reciprocating behavior of former trading partners and whether the personal/impersonal nature of market exchanges can influence the levels of trust and reciprocity that they exhibit. By personal exchange we mean exchange between actors who know one another and by impersonal exchange we mean exchange between actors who do not know one another. In our experiment, subjects interacted with one another as a buyer or seller in a market environment with opportunities to defect and then played the trust game with each subject in the opposite market role. This experimental design allowed buyer-seller pairs to have positive market interactions, where trading partners followed through on the agreements to exchange which they entered, and negative market interactions, where trading partners entered into and ultimately defected on exchange agreements. We utilized a two-treatment experimental design. In the first treatment (which we call Market PE), the market environment permitted exchanges that were more personal (i.e. subjects had a greater opportunity to learn about one another than in the other treatment). In the second treatment (which we call Market IE), the market environment permitted exchanges that were more impersonal (i.e. subjects had less of an opportunity to learn about one another than in the other treatment).

Our results suggest that positive and negative market interactions can affect the trusting and reciprocating behavior of former trading partners. Further, our results suggest that different market institutions can affect how sensitive trading partners’ trusting and reciprocating behavior is to their previous market interactions. In Market PE, people exhibited higher levels of trust and reciprocity to trading partners with whom they had mostly positive market interactions than with whom they had mostly negative market interactions. In Market IE, people exhibited the same levels of trust and reciprocity to trading partners regardless of the nature of their previous market interactions. Stated another way, past market dealings only affected the trusting and reciprocating behavior of subjects who participated in the experimental market where exchanges were more personal but did not affect trust and reciprocity between trading partners who participated in the experimental market where exchanges were more impersonal.

One explanation for this difference is that personal exchanges reveal some relevant tacit and inarticulate information about people that impersonal exchanges cannot and do not. As such, people who participate in personal exchanges are consequently learning about each other [19–24]. Trading partners who engaged in personal exchange may have felt that they had a basis to reward or seek out people with whom they had positive interactions and to punish or avoid people with whom they had negative interactions. Trading partners who engaged in impersonal exchange would have lacked this basis for discriminating between people with whom they had positive or negative interactions. In support of this explanation, we found that despite there being more defections in Market IE than Market PE that only subjects in Market PE exhibited betrayal aversion (i.e. they sort to avoid situations where they might be betrayed).

Before proceeding, it is useful to define the terms we will be using throughout this study. We define trust as the belief held by a person that other people would not betray her and may act in her favor in uncertain or risky situations when they are not expected to do so and when it is not in their interest to do so [25]. Trust is often dichotomized into generalized trust (i.e. trust in strangers) and interpersonal or individualized trust (i.e. trust in known individuals). In this study, we focus on interpersonal trust, as it is what emerges or is eroded when market participants directly trade with one another in market environments. We define reciprocity as a person’s internal motivation to respond favorably or unfavorably to another person’s action (even absent of material gains). Following Fehr and Gächter [26], we say that positive reciprocity has occurred when a person responds cooperatively to a friendly action and say that negative reciprocity has occurred when a person retaliates in response to an unkind or hostile action.

The remainder of the study is structured as follows. The next section (“Markets, trust and social relationships”) discusses some relevant literature on trust and markets. In the section that follows (“Experimental design and procedure”), we explain our laboratory design and procedures. Then, we introduce our hypotheses in “Hypotheses.” In “Results,” we test our hypotheses and present our results. Finally, we discuss our results and offer our concluding remarks in “Discussion and conclusion.”

## Markets, trust and social relationships

It is now well established that markets are dependent on trust [27–28]. Using macroeconomic data, Zak and Knack [29] found a positive correlation between generalized trust, GDP growth and investment levels. Keefer and Knack [30] presented evidence for higher levels of trust in countries with well-established formal institutions that effectively protect property and contract rights and restrict governments from acting arbitrarily (i.e. a dependence of trust on social, economic and institutional contexts in which transactions occur). Similarly, La Porta et al. [31] showed that the proportion of trusting people was negatively correlated with inflation rates and positively correlated with GDP growth across countries. Likewise, Guiso et al. [32] found that greater bilateral trust between two countries is associated with higher volumes of trade between the countries. Here, bilateral trust is affected not only by the specific characteristics of the country being trusted but also by the cultural traits shared by the two countries (religious, genetic and somatic similarities, and history of conflict). Torsvik [33] also argued that patterns of horizontal association between individuals in a community can produce the types of trust that can reduce transaction costs in the economic sphere, which in turn boosts social cooperation. And, Chamlee-Wright [13] and Ingram and Roberts [17] demonstrated how certain profiting opportunities were only available to individuals who shared with strong and tight bonds with others in their industry. Furthermore, higher trust societies are also associated with efficient judicial systems [34], high-quality government bureaucracies [35], less government intervention [36], less corruption and better financial markets [31, 37], less crime [38] and better health [39]. Admittedly, some scholars, such as Portes and Landolt [40] and Annen [41], were less enthusiastic about the impact of social relations on economic outcomes and highlighted how they might impede economic performance.

It is important to note that trust plays an important role in facilitating market interactions in a variety of market contexts. Indeed, trust can be important within business organizations, between businesses, between businesses and their customers, and in supporting the social context in which market interactions take place. However, rather than focusing on the importance of trust for markets, this study examines if markets can engender trust. This study, thus, contributes to (a) the literature on markets and social relationships, (b) the general experimental economics literature on the determinants of interpersonal trust and (c) the narrower experimental economics literature on the effect of market settings on interpersonal trust.

### Markets and the development of social relationships

There appears to be something of a consensus that social bonds can, at least occasionally, develop in markets. There is, however, nothing like a consensus concerning the quality of these commercial friendships. Moreover, there is wide disagreement concerning whether or not markets are more likely to promote or disrupt social bonds.

This ambiguity over the quality of the social bonds that develop between market actors and the likelihood that these social bonds will emerge has arguably always characterized discussions surrounding the connection between markets and sociability. Smith [42], for instance, believed that markets have the potential to both disrupt and enhance social bonds. Specifically, he believed that the growth of markets led to a weakening of familial bonds while at the same time creating an opportunity for the development of deep social connections between co-workers. For instance, Smith [42, p. 223] argued that,

[i]n commercial countries, … the descendants of the same family, having no such motive for keeping together, naturally separate and disperse, as interest or inclination may direct. They soon cease to be of importance to one another; and in a few generations, not only lose all care about one another, but all remembrance of their common origin, and of the connection which took place among their ancestors.

As countries transitioned from pastoral societies to commercial societies, Smith believed, familial bonds became less important. Smith [42, p. 224] also believed, however, that “[c]olleagues in office, partners in trade, call one another brothers; and frequently feel towards one another as if they really were so.” Commercial relationships, he believed, often transform into social friendships characterized by the trust, warmth, affection and mutual accommodation that we expect of friendships [43–45].

Others have, of course, taken a less ambiguous view of the effect that markets have on social relationships. Marx, Polanyi and Weber, for instance, all believed that market relations and social relations were necessarily at odds and that the expansion of market activity (if unchecked) would have a deleterious effect on community. Recall, Marx [46] claimed that there was an unavoidable antagonism between employers and employees. He [47] also argued that market activity was inherently alienating, leaving man estranged from other men. And, according to Marx [48, p. 49], “[t]he greater and the more articulated the social power is within the relationship of private property [i.e. the greater the scope of market exchange relations], the more egoistic and asocial man becomes, the more he becomes alienated from his own nature.” Similarly, Polanyi [49, p. 3] warned that the growth of the “self-regulating market” risked “annihilating the human and natural substance of society.” Additionally, Weber [50] believed that market relations and fraternal bonds were at odds with one another. “The market community,” Weber [51, p. 76] wrote, “is the most impersonal relationship of practical life into which humans can enter with one another.” And, “where the market is allowed to follow its own autonomous tendencies, its participants do not look toward the persons of each other … there are no obligations of brotherliness or reverence, and none of those spontaneous human relations that are sustained by personal unions” [51, p. 76].

Elsewhere, communitarians have worried about the expansion of the market into other social realms and how it undermines moral values on which community relies [52–55]. In a market society which is based on economic actors pursuing their own economic ends, once friendly society members turn into competitors and, thus, social bonds yield to rivalry as selfishness becomes the dominant value. Gray [56, p. 36], for instance, lamented that “[t]he unintended consequences of policy of freeing up markets was a fracturing of communities, and a depletion of ethos and trust within institutions, which muted or thwarted the economic renewal which free markets were supposed to generate.” Similarly, Gudeman [57] believed that the market corrupts social relationships. As the market expands, Gudeman argued, the community will shrink as people increasingly spend more time in the economic rather than the communal sphere, pursuing material desires and spending less time building meaningful social relationships. Markets, according to these scholars, are incapable of engendering social relationships based on trust and reciprocity and, without intervention, will destroy values that we treasure as a society.

Relatedly, Putnam [39] acknowledged that workplace ties can and do develop but remains skeptical that these ties can be as strong as ties developed in other settings. Friendships formed at the office, he argued, can be a substitute for friendships developed elsewhere. According to Putnam [39, p. 87], “many people form rewarding friendships at work, feel a sense of community among coworkers, and enjoy norms of mutual help and reciprocity on the job.” However, he did not believe that the possibility that co-workers can develop friendships makes up for the loss of social connections that has occurred because of the changes in the nature of work and the technological developments that resulted from the growth of markets. According to Putnam [39, p. 87], there was “no evidence whatever that socializing in the workplace, however common, has actually increased over the last several decades.” Additionally, Putnam [39, p. 87] insisted, social connections formed in the workplace are inferior to connections formed in other settings and “tend to be casual and enjoyable, but not intimate and deeply supportive.”

Zelizer [58] described the view that markets and community are separate spheres that often impinge on and occasionally destroy one another as a “hostile worlds” view. This view, she explained, does not do justice to the connected lives that human beings experience where markets and community are constantly enmeshed and often reinforce one another. Similarly, Granovetter [8] argued that social action can be economically conditioned, and that market activity can lead to the development of meaningful social connections. As Granovetter [8, p. 495] acknowledged, “business dealings [sometimes] spill over into sociability … especially amongst business elites.” Additionally, in explaining why consumers tend to trust and prefer their “own past dealings” as sources of information, Granovetter [8, p. 490] noted that “individuals with whom one has a continuing relationship have an economic motivation to be trustworthy” and “continuing economic relations often become overlaid with social content that carries strong expectations of trust and abstention from opportunism.” Repeated successful interactions in the market, for Granovetter, not only serve as a foundation for future market transactions but can also be a basis for social friendships.

Storr [12], however, viewed the market as a social space where meaningful conversations beyond the bid-ask take place and where meaningful social relationships beyond exchange and competition occur. “The market,” Storr [12, p. 148] explained, is “a social space where people form friendships, meet their husbands and wives, and connect with their parents, children, and siblings.” Indeed, a number of studies demonstrated the variety of social relationships that can emerge in markets. Duneier [10] and Anderson [11], for instance, described how commercial spaces like restaurants and bars serve as important gathering spots for the residents of urban areas. Likewise, mentor-mentee relationships frequently develop into close friendships or even into relationships that have some of the characteristics of parent-child relationships. According to Kram [59, p. 614], these relationships, typical in several trades and professions, can fulfill a number of “psychosocial functions including role modeling, acceptance-and-confirmation, counseling, and friendship.” Similarly, certain seller-customer relationships frequently develop into deep friendships. It is not uncommon for lawyers and their clients, hairdressers, barbers and their customers, and retailers and their shoppers to become quite close. “Commercial friendships, similar to other friendships,” Price and Arnould [16, p. 50] wrote, “[can] involve affection, intimacy, social support, loyalty, and reciprocal gift giving.” The social relationships that develop between co-workers can, of course, range from acquaintanceships to social friendships. While work acquaintanceships seem to be more common than social friendships between coworkers, Argyle and Henderson [60] explained that friendships between coworkers who interact socially outside the workplace do frequently develop. Similarly, Bridge and Baxter [61, p. 200] wrote, “for many adults who work outside the home, friendships frequently evolve from existing role relationships in places of employment and are maintained within those organizational settings.” Berman et al. [62, p. 219] likewise found that “workplaces often have features that may facilitate friendship making. Workplaces are sites where people meet others, including co-workers, clients, members of other departments or organizations, and supervisors.”

Discussions of relational goods are also relevant here [63–64]. Gui [65], for instance, argued that the personal encounters that individuals experience in the market are opportunities for the joint production of relational goods (i.e. intangible valuable goods associated with relationships) with the other parties to the exchange. Although these goods are traditionally defined as intangible goods that arise out of the relationship between individuals and can only be consumed if certain known others jointly act to acquire it, they can also describe non-instrumental interpersonal relationships [66]. Mota [66], however, asserted that enhanced market competition means less relational goods. Similarly, Becchetti et al. [67] suggested that relational goods will tend to be underprovided and under-consumed in the market.

Although the literature described above does acknowledge the potential of commercial relationships to morph into social relationships, there has been no consensus regarding whether or not the impact of markets on social relationships will, on net, be positive or negative. Moreover, the conditions under which commercial dealings are likely to morph into social relations and the conditions where they will support or disrupt social bonds remain underexplored. Furthermore, as will be discussed below, efforts to explore the link between markets and social relations characterized by interpersonal trust have tended to focus on how markets depend on, rather than generate, trust. Our study attempts to fill this gap in the literature.

There is, of course, a literature in business marketing and management that looks at how businesses can intentionally build trust. Specifically, this literature examines the role of trust in buyer-seller relationships and how businesses could utilize these relationships in their marketing techniques and sales strategies to reach their customer base. An exhaustive review of this literature is, obviously, beyond the scope of this project but the research referenced below should give a sense of how that literature might relate to this project. Dwyer et al. [68], for instance, highlighted the pivotal role that trust plays in buyer-seller relationships and proposed a model of buyer-seller relationship development in marketing. They postulated that relationships evolve through five general phases (awareness, exploration, expansion, commitment, and dissolution) and explained that each phase represented a major shift in how the buyers and sellers treated one another. In this model, trust begins building towards the end of the second phase (exploration), as relational expectations are developed and calibrated between a pair of market participants. Trust within a relationship continues to grow in the third phase (expansion) as the pair increasingly grow interdependent on one another and see continual increases in economic gains and in the fourth phase (commitment) as economic, communication, and/or emotional resources are exchanged. A relationship between a buyer and a seller exists so long as the relationship is not strained and both parties continue to wish to be a part of the relationship. In short, Dwyer et al. speaks to how prior experience with particular trading partners provides market participants the opportunity to build long-term relationships based on credulity and trust.

Batt [69] also investigated how people navigate and conduct business in an environment of general distrust and disputation. Over the years, as major supermarkets increasingly dominated sales in the fresh produce industry in Perth, Australia, the market moved from auctions to private negotiations as the primary way of conducting business. Batt observed that, with the absence of the auction, a great deal of distrust has emerged between the growers and the market agents (i.e. the intermediaries who purchase produce on behalf of retailers). “Unlike the auction, where the price at which the produce is sold is public knowledge and, to some extent, where the identity of the buyer is revealed,” Batt [69, p. 66] explained, “the lack of transparency inherent in private negotiations has resulted in an underlying atmosphere of distrust between growers and market agents.” He found that a grower’s satisfaction with a transaction had the most significant influence on building trust between the said grower and their most preferred market agent. Specifically, the growers expressed being most satisfied when (1) they believed that the market agents treated them fairly and equitably; (2) they felt as though their expectations of high returns had been met; and (3) they felt adequately rewarded for their efforts. This study points to the potential of positive market interactions generating trust, especially when market agents make “various relationship-specific investments” [69, p. 75].

To the best of our knowledge, there are only a few meta-analyses that investigate the role of trust in relationship management and marketing channels (i.e. networks/systems of people and organizations that get products from the point of production to the point of consumption) [70–71]. Of them, Geyskens et al. [72] is closest to our topic of investigation here. Geyskens et al. performed a meta-analysis on 24 major studies that empirically investigated the antecedents and/or consequences of trust in marketing channels. Using a type of categorization method, they found that trust shared strong, robust, and heterogeneous correlations with seven other channel relationship constructs. Their analysis demonstrated that trust shares the strongest correlation with sentiments, followed by actions, performance, channel decision structure, environmental uncertainty, channel decision influence patterns and power/dependence patterns. “These findings,” said Geyskens et al. [72, p. 242],

suggest that when building trust is an important organizational goal, managerial focus on sentiments (such as goal compatibility and fairness), action (such as communication, opportunistic behavior, and support) and economic outcomes may be most effective. In other words, relationships are not prisoners of the environment and power structure, but whether trust develops depends on how parties feel and behavior and on the outcomes developed.

The contemporary marketing research on buyer-seller relationships, as we understand it, is principally concerned with identifying the factors that make for good relationships and understanding how buyers and sellers leverage these relationships for future market interactions and earnings. (See also Han et al. [73], Smeltzer [74], Zaheer et al. [75], and Hill et al. [76].) While related, this is distinct from our aims in this study. Here, we investigate the process by which those who are initially strangers come to share relationships of trust and reciprocity in market settings.

Perhaps more relevant to our project is the substantial body of research by members of the Industrial Marketing and Purchasing (IMP) Group (see, for instance, Håkansson [77] and Håkansson and Snehota [78] for an overview). This literature considers markets in terms of the “atmospheres" in which relationships between industrial firms are developed and maintained. Specifically, relationship atmospheres are the contexts where business relationships are formed and maintained, including their emotional settings, the formal and informal rules governing those relationships and the perceptions of as well as past experiences with business relations (Hallén and Sandström [79]). As Cunningham and Turnbull [80, p. 306] explained,

The establishment and maintenance of inter-company relationships is through the contacts of key individuals. The behaviour of the individuals acting either in their own right, or as representatives of their companies, creates the “atmosphere” in which inter-company relationships occur. An atmosphere of trust or deceit may be created, an atmosphere of brutal exercise of power or restraint may be built up and developed by the actions of individuals.

Atmospheres, in turn, become the sites where transactions between firms occur. They are both the products of past dealings and the environments in which future dealings occur (Sutton-Brady [81]). Atmospheres are, thus, key factors in the development of interfirm business relationships as well as the nature and characteristics of these relationships. Scholars within the IMP Group network [82] developed this insight into a widely used framework for discussing relationship atmospheres that involves classifying atmosphere across multiple dimensions: power versus dependence, trust versus opportunism, closeness versus distance, cooperation versus conflict/competitiveness, understanding and commitment (see, for instance, Hallén and Sandström [79]). Although this body of work has proven to be a fruitful frame for considering the determinants of interpersonal trust that we explore here, the atmosphere created within the market settings in our experiment differs significantly from the atmospheres in “industrial markets [which] are characterized by stability instead of change, long lasting relationships instead of short business transactions and closeness instead of distance” [79, p. 6]. Additionally, the studies inspired by the seminal edited volume by the IMP Group [79] have tended to rely on interviews, surveys or other similar empirical approaches rather than experiments.

Unlike other empirical strategies, however, our experiment gives us the unique advantage to isolate and study one specific aspect of relationship-building–positive and negative market exchanges. Furthermore, our design and empirical strategy allow us to quantify and compare relationships of trust with relationships of distrust and no trust, which is largely omitted in marketing literature, as well as sociology and economics literature discussed above. Additionally, while the literature reviewed above points to the potential and limits of trust developing between actors in a variety of commercial settings and through several different types of market interactions, our experiment focuses on the type of interaction where relationships characterized by trust and reciprocity are perhaps least likely to occur (i.e. between buyers and sellers with limited to no histories with each other and no likelihood of future interactions qua their experimental identities beyond the experiment).

### The determinants of interpersonal trust

There is now a considerable literature within experimental economics on trust and trustworthiness and on the determinants of interpersonal trust and reciprocity (which is largely considered to be interchangeable concepts by the field). The trust game designed by Berg et al. [83] is a two-player economic game where the players make decisions sequentially (i.e. one-by-one) and is the most popular tool to measure (interpersonal) trust in experimental economics. The trust game simplistically portrays a situation where a person must take a risk in trusting another person without knowing with certainty whether or not the other person would repay the trust she was shown and whether or not the other person is thus worthy of said trust. Because the original trust game quantifies the trust and repaid trust (commonly interpreted as trustworthiness and reciprocity in experimental economics) using monetary exchanges or transfers, it is also sometimes called the investment game. Johnson and Mislin [84] conducted a meta-analysis of Berg. et al.’s [83] trust games performed around the world, involving 162 replications of the trust game and more than 23,000 subjects. Their analysis revealed several key findings. First, playing with real (as opposed to simulated or computerized) counterparts significantly impacts trusting and reciprocating behavior and is associated with higher transfers. Second, investors in Africa transferred the least compared to their North American counterparts, followed by Asians, South Americans and Europeans. This regional heterogeneity corroborates the main finding from research on generalized trust using macroeconomic data (on which we discuss further in the next section). Third, Johnson and Mislin found that student populations sent back significantly fewer tokens than non-student populations, reinforcing the notion that younger people reciprocate less and are less generous than older people [85–87].

Some efforts have probed into the limitation of this trust measure. Cox [88], for instance, demonstrated that trust, as measured using the trust game, confuses trust with altruism. Bohnet and co-authors [89–90] as well as Aimone and Houser [91–92] argued that it confounds trust with betrayal aversion. Still more studies showed that the measurement omits important facets of trust, such as risk attitudes [93–94], beliefs and attitudes [95], financial health and marital status [96], physical health, employment status, household size and political views [97], education [98], age [99] and gender [100]. Moreover, trust and reciprocity appear to be positively associated with levels of oxytocin [101–102].

Furthermore, numerous studies have found links between trust/reciprocity and social distance/familiarity. For instance, Foddy and Yamagishi [25] found that in-group favoritism derived from a social expectation of generalized reciprocity leads individuals to trust strangers from their own group at higher levels than out-group members. Habyarimana et al. [103] observed that in-group favoritism in trust contexts does not appear to be based on a preference to interact with similar people but rather based on norms of cooperation reinforced by group members (which reduced the likelihood of exploitation). Barr [104] reported that people residing in resettled communities in Zimbabwe (where the networks defined by kinship were quite sparse) transferred less as Player 1s in the trust game than those in traditional communities (where such networks were dense). Barr et al. [105] found that social network centrality is another source of differential trust. They conducted field experiments in Africa and found that social network position, controlling for sociodemographic variables, has a positive effect on trustworthy behavior; the more central a person’s position in the social network, the more likely they are to act trustworthily. “[T]rusting behavior is partially based on expectations about people’s trustworthiness,” they [105, p. 617] concluded. In fact, Barr et al.’s findings are consistent with and are explained using Burt’s [106–107] work on brokerage. Additionally, Glaeser et al. [108] found that trust and trustworthiness grow the closer individuals are socially and that race, nationality and social status can affect trustworthiness. There have also been efforts to determine the impact of institutions on trust [109–110] as well as cross-country comparisons of trust [31, 84, 111–112]. Trust also appears to be endogenous to a region’s institutions [27, 113].

Despite the considerable efforts to identify the determinants of trust and trustworthiness, there are relatively few studies on how engaging in markets affects measured levels of trust and trustworthiness. This is somewhat surprising given the important and strong relationship between trust and economic performance.

### Market settings, generalized trust and interpersonal trust

Markets seem to engender trust and prosociality. Several studies have suggested that there is a positive link between an individual’s exposure to markets and their performance in laboratory experiments that measure trust, reciprocity, social cooperation and altruism. Henrich and coauthors [114–115], for instance, concluded that market integration explains a large portion of the behavior variation across societies that are observed in economic experiments. The more the market is integrated into a community the higher levels of prosociality they exhibit in ultimatum games. Tracer [116] also found that there was some (albeit weak) support for the notion that a greater level of market integration at the community and individual level leads to greater prosociality. Likewise, Ensminger [117] found that exposure to markets was a predictor of offer size within ultimatum and dictator games. In fact, Ensminger and Cook [118] added, a rural American population displayed the highest level of prosociality including trust and trustworthiness among the various small-scale communities in their sample. (To provide additional context, this rural American population was the only community in a fully market-oriented society in their cross-country sample.) Henrich et al. [119] observed that market exposure was positively correlated with other-regarding preferences. Tu and Bulte [120] explored the links between trust and market integration and concluded that trust (as measured using a trust game) is positively associated with labor market participation. Additionally, Herz and Taubinsky [121] showed how market experience matters in shaping fairness preferences *ex post*.

Furthermore, Fehr and List [122] observed a significantly higher display of trust and trustworthiness by coffee mill CEOs than students in their experiment in Costa Rica. They [122, p. 764] concluded that “[their] results indicate that nonpecuniary motives may play a more important role in transactions among CEOs than in transactions among students” and that “CEOs better recognize the vital role that trust plays in eliciting trustworthy behavior [compared to students].” And, the mere thought of the positive aspects of markets and exchange can induce people to be more trusting and to hold higher beliefs about the trustworthiness of strangers. Al-Ubaydli et al. [123] implemented a two-treatment design where subjects performed a priming task followed by the trust game. Their priming task asked subjects to form grammatically correct sentences using 4 words from a list of 5 randomly arranged words. (For example, an acceptable answer to the random list <flew, eagle, the, plane, around> was <the eagle flew around>.) In their treatment group, the priming task asked subjects to form grammatically correct sentences involving the market and trade. This method, the authors argued, led subjects to think about markets and trade without their awareness. In their treatment group, 12 out of 15 questions involved sentences about trade and markets. In their baseline group, all 15 questions did not include sentences about trade and markets. They concluded that market-priming significantly increased the amount sent by the senders/investors to anonymous partners. Furthermore, they verified that this increase was a consequence of increased trust rather than an increase in altruism using Cox’s [88] design.

Not all of these studies, however, concluded that markets can increase trusting and trustworthy behavior in laboratory experiments. Gurven [124] concluded that differential market exposure was not an important influence on the behavior of Tsimane in ultimatum and public goods games. Similarly, Bowles [125], Hoffman et al. [126], Schotter et al. [127] as well as Reeson and Tisdell [128] found that the more aspects of the laboratory experiment resemble a market, the less likely participants are to display other regarding preferences. Similarly, Falk and Szech [129] found that market exchange and negotiations can decay moral values. Xin and Xin [130] utilized data on interpersonal trust in China from 82 studies and found a negative correlation between trust and market economy development.

Although there are multiple studies that link markets and prosocial attitudes, whether or not markets will tend to promote trust that carries over into non-market settings remains an unsettled question. Collectively, however, these studies do suggest that having more familiarity, experience, and exposure to markets affected how people treat and behave towards one another. However, to the best of our knowledge, in a majority of the studies in this literature, with the exception of Herz and Taubinsky [121], the degree to which subjects were integrated into a market was an exogenous characteristic that the subjects brought into the experiment, or a mode of thinking that was primed within the experiments; these studies did not have their subjects engage in a market experiment before examining their prosocial behavior. Instead, they tended to use demographic, survey or other data to determine the degree of market integration or to introduce certain aspects of modern markets (like anonymity or exchange) into their experimental design. There, thus, remains an opening in the literature to explore the endogenous formation of social bonds characterized by trust and reciprocity in market settings using laboratory experiments.

### Can markets promote trust and reciprocity? If so, how?

Again, our main contention is that markets are important for the development of meaningful social bonds. Engaging in market transactions gives individuals an opportunity to learn about and develop relationships characterized by trust and reciprocity with other market participants. These relationships are especially likely in institutional settings that permit personal exchange and can then carry over into non-market settings.

The general hypothesis that we explore here can be stated as follows:

H: Individuals who have developed a positive relationship with someone within a market setting will exhibit higher levels of trust and reciprocity when interacting with that person outside of a market setting than when interacting with individuals with whom they have developed a negative relationship within a market setting.

A positive relationship developed in a market setting, for our purposes here, is one where trading partners kept their commitments with each other more often than not.

There are at least two mechanisms through which markets where personal exchange is possible might promote meaningful social bonds characterized by trust and reciprocity. First, markets where personal exchange is possible might give individuals an opportunity to learn about specific others. Stated another way, certain kinds of markets give individuals an opportunity to reveal themselves to be trustworthy or not. Consequently, within these market settings, individuals can learn whether or not someone is a fair dealer and a promise keeper and can deliberately choose to reveal themselves as trustworthy or untrustworthy individuals. This knowledge about the nature of specific trading partners would then likely impact future interactions with them both inside and outside the market. Of course, why someone breaks their promise might matter (i.e. they might have changed their mind about the deal or might want to cheat their trading partner). Regardless of the reason that someone broke their promise, that they broke their promise does reveal something about the promise breaker. Second, markets might give people an opportunity to learn about others in general. The more people have positive experiences in the market, the more positive relationships that they develop with specific others in a market setting and, consequently, the more likely they are to trust and to reciprocate the trust of others. In other words, successful dealings in the market might condition individuals to the possibility of mutually beneficial exchanges and, so, promote the development of pro-social attitudes.

We believe that a laboratory experiment where individuals engage in a market game with the possibility of defection followed by a trust game will be able to show that markets can promote the development of meaningful social bonds characterized by trust and reciprocity. We also believe that comparing results from a treatment where more personal exchange is permitted and a treatment where more impersonal exchange is permitted will help us establish the channel through which market interactions are impacting trust and reciprocity.

## Experimental design and procedure

Our experiment consisted of two treatments. We first provide a short overview of our experiment and treatments before we discuss our treatments in detail.

In both treatments, subjects first played a market game, followed by a trust game. In the first treatment, we employed a bilateral, simultaneous offer market game, which was based on the Chamberlin market [131]. In the second treatment, we utilized a version of the double auction market where buyers and sellers simultaneously submitted offers (in the form of bid and ask prices). Our experiment was programmed in z-Tree [132].

Our bilateral, simultaneous offer market game differed from our modified double auction market game in one important way. The bilateral, simultaneous offer game permitted market participants to identify, select and privately negotiate with specific trading partners. Additionally, market participants did not know what offers were being made outside those that they personally sent and received. In the modified double auction market game, however, market participants could not choose with whom to negotiate. Instead, the offers were publicly posted and, so, every market participant observed every bid and ask price within the experiment. This key difference between the two market games allowed us to shape how the subjects experienced their interactions with specific trading partners–notably, how personal the market interactions with particular trading partners seemed to our subjects. We believe it is uncontroversial to suggest that having the ability to privately negotiate with someone of one’s own choosing can facilitate interactions that are more personalized and that not having the ability to privately negotiate can hinder the degree to which these interactions are personalized. This was the only difference between our two treatments.

Our two treatments shared some similarities. First, the buyers and sellers were identifiable to each other. We informed the subjects that they would be assigned to an experimental identity at the beginning of the experiment and that everyone would be identifiable by these identities throughout the experiment. Second, unlike the original Chamberlin market and the standard double auction, information about successful agreements (specifically, the agreed trading price) and executed trades were not announced nor shared with other market participants at any point. The trading price on which a buyer and a seller agreed remained private to the involved buyer and seller. Third, information about endowments was also private knowledge.

Below, we first describe the treatment with the bilateral, simultaneous offer market game that allowed exchanges to be more personal (Market PE). Then we describe the treatment with the double auction, which allowed exchanges to remain more impersonal (Market IE). Again, the only difference between Market PE treatment and Market IE treatment in terms of design was how the subjects negotiated trading prices (i.e. the first stage of the market game).

### Market PE treatment

The computer randomly assigned subjects to a market role at the beginning of the market game. Each market consisted of four buyers and four sellers. The computer endowed each buyer with a budget, randomly selected from a uniform distribution between 11 and 100 experimental dollars (E$), and each seller with one unit of a good with a cost of E$10. These buyers and sellers interacted with one another for ten trading rounds.

A trading round comprised of two stages: (1) the negotiation stage and (2) the defection stage. In the first stage, subjects interacted in the market and negotiated agreements with whomever they wished in the opposite market role for two and a half minutes. Specifically, a buyer (seller) picked one trading partner out of four possible sellers (buyers) in each round and proposed a price by sending an offer to this chosen trading partner. The seller (buyer) who received the offer then had the choice to accept, reject, or ignore the offer, or to send a counteroffer.

We did not constrain the buyers (sellers) with regards to how many sellers (buyers) they negotiated with in each trading round. However, we did restrict subjects to negotiating and trading with only those in the opposite market role, thereby preventing collusions between the four buyers or the four sellers from emerging. Furthermore, we restricted each subject to maintaining one open offer at a time. This meant that a subject could not make a new offer to the same or different trading partner until the offer she already sent had been accepted or rejected by the trading partner, or withdrawn by herself. Consequently, each subject could only send one offer at a time, but could have received multiple offers from multiple potential trading partners.

When an offer was accepted, the involved parties exited the market and any remaining active offers either sent or received by them automatically expired. Any subject who did not successfully enter an agreement with a trading partner by the end of the two and a half minutes earned E$0 for that trading round.

Once the negotiation stage ended, those buyers and sellers who successfully entered agreements moved onto the second stage of the market game. In this second stage, the subject who sent the accepted offer (henceforth, the Proposer) had a choice between executing the agreement as made during the negotiation stage or defecting on said agreement. If the Proposer decided to execute the agreement, the involved buyer and seller earned profits calculated in the standard manner, and the buyer received the good. If the Proposer decided to defect on the agreement, she took both the good and the cash (i.e. the agreed trading price) while the Recipient (i.e. the subject who accepted the Proposer’s offer) earned nothing for that trading round. More specifically, if the Proposer was a seller, she earned the experimental cash equivalent to the agreed trading price plus E$10 (i.e. the experimental cash value of the good that she had retained). If the Proposer was a buyer, she earned the experimental cash equivalent to her endowed budget plus E$10 (i.e. the experimental cash value equivalent to the good she had received). While the Proposer made the execute/defect decision, the Recipient was informed by the computer what her earnings would be if the Proposer executed and defected on their agreement. The trading round ended once all of the Proposers submitted their execute/defect decisions. The computer then privately displayed each subject’s round profit, the identity of her trading partner (if applicable) and her respective Proposer’s execute/defect decision (if she was a Recipient). A new trading round began with the computer endowing the buyers with new budgets and the sellers with one unit of good each.

As a clarification, information about any sent and accepted offers in the negotiation stage, and about any executed and defected agreements was known only to the involved parties and was kept private from other subjects in the experiment. Also, the computer did not display any information about executed and defected agreements in past trading rounds. We utilized record sheets to allow subjects to carry their own market histories throughout the experiment, which we further discuss further later in this section.

After the market game, the subjects played a series of one-shot trust games with a multiplier of three [83]. At the beginning of the trust game, the computer systematically assigned buyers to a trust game role and the sellers to the other trust game role. Like Berg et al. (Ibid.), we endowed both Players 1 and 2 with ten tokens each at the beginning of the trust game. First, Player 1 made a decision on how to split her endowment with Player 2 (henceforth denoted as *x*). Then, Player 2 made a decision on how much of Player 1’s tripled transfer (henceforth denoted as 3*x*) she wanted to send back to Player 1 (henceforth denoted as *y*). The trust game ended here, once all of the Player 2s submitted their transfer decisions, with Player 1 earning 10 − *x* + *y* tokens and with Player 2 earning 10 + 3*x* − *y* tokens. In our experiment, each subject played a total of four trust games in their assigned trust game role. More specifically, each buyer (seller) played a trust game with each seller (buyer) and made all four transfer decisions simultaneously. So, when the computer displayed trust game results at the end of the trust game, the computer privately displayed each subject’s own transfer decisions, her four trust game partners’ transfer decisions and her token earnings with each trust game partner. As in the market game, decisions and results of each trust game were only revealed to the involved Players 1 and 2 and were kept private from the other subjects in the experiment.

The experiment concluded after the trust games. We conducted post-experiment surveys through which we gathered basic demographic information about the subjects and a risk attitude measure [133]. For this risk attitude measure, we replicated Holt and Laury’s [133] low-stake measure (i.e. the safe lottery with $2 and $1.6 and the risky lottery with $3.85 and $0.10). We do not report findings from the risk attitude measure in this study.

### Market IE treatment

To reiterate, Market IE was identical to Market PE in all aspects except for the way in which subjects negotiated trading prices in the first stage of the market game.

In each trading round, once the market opened, buyers and sellers submitted bid and ask prices that were publicly posted for everyone in the experiment to see. Subjects could only accept a posted offer and could not withdraw their offers (or reject others’ offers), but could submit as many offers as they would like. Once a buyer (seller) accepted a posted ask price (bid price), the involved parties exited the market and any remaining active offers submitted by them automatically expired. Any subject who did not successfully enter an agreement with a trading partner by the end of the two and a half minutes earned E$0 for that trading round.

Because we are principally concerned with buyers and sellers making personal connections with one another, we did not allow the computer to automatically enter a buyer and seller into an agreement even when their bid and ask overlapped as occurs in the standard double auction experiments. Specifically, an agreement did not automatically occur when a seller submitted an ask price that was less than or equal to the highest posted bid price or when a buyer submitted a bid price that was greater than or equal to the lowest posted ask price.

Like the market in Market PE, we restricted subjects to trading with only those in the opposite market role; in other words, buyers (sellers) could see what offers other buyers (sellers) were proposing, but could not accept their offers or attempt to collude with them in any way. Again, information about successful agreements and about executed (and defected) trades was only known to the involved parties and was not shared with other subjects in the experiment at any point.

### Procedure

This study was approved by the Office of Research Integrity & Assurance at George Mason University (Project Number: 477726–4). All 132 subjects signed a written informed consent form prior to participating in the study.

The experiment was conducted between October 2013 and April 2015 with George Mason University students who did not have prior experience with a trust game environment. Subjects were randomly selected from a pool of registered students by the recruitment system to receive email invitations to participate in our experiment. Every subject signed a written consent form prior to participating in the study. They were seated in individual and partitioned cubicles (so that they could not observe others’ actions) upon arrival. We read the instructions aloud prior to the market game and gave subjects opportunities to privately ask us questions about the instructions. Afterward, we administered comprehension quizzes. We incentivized the market game comprehension quiz and the subjects received $2 if they completed the quiz correctly on their first try. After everyone completed the quiz, the subjects went through a practice round prior to the ten trading rounds to familiarize themselves with the market game interface. (We did not offer any feedback after the practice round before they began the first trading round.) The computer assigned each subject an experimental identity immediately prior to the first trading round, by which they were identifiable by other subjects throughout the experiment.

After the last trading round of the market game, we distributed copies of the trust game instructions, which we read aloud, and gave subjects opportunities to privately ask us question on the instructions. However, with the trust game, the subjects did not have a practice round and immediately played the trust games after everyone completed the comprehension quiz. The quiz for the trust game was not incentivized.

At the end of the experiment, each subject privately rolled a series of ten- and four-sided die to randomly select a trading round, a trust game and a lottery choice, and was privately paid according to their decisions/outcomes. Experimental dollars were converted at the exchange rate of E$2.5 to $1 and tokens were converted at the exchange rate of three tokens to $1. All subjects received $5 for arriving on time.

On average, both treatments lasted 120 minutes. Our sample included a total of 132 subjects and included four graduate students, 71 females, 26 non-US citizens, 58 subjects who self-identified as being “white/Caucasian” and 6 undergraduates who reported being economics majors. None of the subjects were previously acquainted with the authors. The average age of our subjects was 20.97 years. The average total payment was $22.55 and ranged from $6 to $57 (inclusive of the on-time and quiz fees).

### Defining buyer-seller relationships

People will tend to experience both positive and negative interactions as they encounter various people during the course of their lives. In reality, it is possible for Person A to have generally pleasant interactions with Person B, but have generally unpleasant interactions with Person C. It is entirely possible for Person D to simultaneously have generally unpleasant interactions with Person B. In addition, we tend to treat people dissimilarly depending on our assessments of them which, more often than not, have been influenced by (but not limited to) our interactions with them. For instance, we are more likely to be nicer and to be more willing to give someone the benefit of the doubt if we have had generally pleasant interactions with her but not if we have had generally unpleasant ones. In this study, because we are interested in the trust and reciprocity that people exhibit towards a specific person after they have interacted in the market (i.e. after they have “gotten to know them”), we investigate the history of each buyer-seller relationship, not each subject’s history, in the market in this study.

We say an ***agreement*** has been reached when a Recipient accepts a Proposer’s offer in the first stage of the market game. We describe an agreement that was executed by the Proposer in the second stage of the market game as being positive and label it as a ***positive interaction***. Similarly, we describe an agreement on which the Proposer defected in the second stage of the market game as being negative and thus label it a ***negative interaction***.

In order to investigate the trust and reciprocity that develop between a specific buyer and a specific seller in the market environments, we analyze how subjects’ perceptions about specific trading partners and the relationships that they share affected their trust game decisions. We categorize a buyer-seller pair by whether the pair had generally positive or negative interactions and do so by calculating the proportion of defected agreements within their relationship. More specifically, let ***p_ij_*** denote the proportion of agreements reached between *buyer i* and *seller j* where *i*,*j* ∈ [1,4] and on which *buyer i* or *seller j* then defected as the Proposer in the second stage of the market game. We define a relationship between *buyer i* and *seller j* as a ***positive (trading) relationship*** if *p_ij_* < 0.5. We define a relationship between *buyer* *i* and *seller* *j* as a ***negative (trading) relationship*** if *p_ij_* ≥ 0.5. A buyer and a seller who never once reached an agreement with each other across the ten trading rounds are defined as having ***no (trading) relationship***.

It seems intuitive to expect people’s decisions regarding how much trust and reciprocity to place in a known associate to depend on their overall experiences with their associates. This is how we analyze our data throughout the study. However, a person could assess the nature of their relationship with another in two other ways: by her first impression of her trading partner and by how their relationship ended (i.e. their last interaction). We analyze our data using these alternative definitions of positive and negative relationships in the appendix (“Supporting Information”). Our results using negative and positive relationships defined by first impressions and last interactions are consistent with our results using negative and positive relationships as defined here.

Given the importance of past market interactions for our analysis, we wanted a way for subjects to carry their own market histories to the trust game without drawing undue attention to it. Before the first trading round began, we provided subjects with record sheets on which we asked them to record their experimental cash earnings and their trading partners’ identities (if they reached an agreement with someone) after each trading round. To mask the importance of their market histories, we also provided the subjects with another set of record sheets immediately prior to the trust games, on which we asked subjects to record their token earnings alongside their trust game partners’ identities. Subjects were told that these record sheets would be used in the final cash payment process.

In this study, we are chiefly interested in understanding how a person’s *perception* about a business partner and about the relationship that they share affects the amount of trust and reciprocity she exhibits to the said business partner. For this reason, we considered the information on executed agreements as reported by subjects (on their record sheets), not those recorded by the computer, to be more truthfully reflecting their subjective market experiences. As such, we report results from analyzing information from record sheets in the results section. By this logic, we opted to utilize and analyze information about executed agreements on the subjects’ record sheets whenever there was a discrepancy between a subject’s record and the computer’s record.

A number of subjects misreported their trading partner’s identities on their market record sheets, thus affecting the total number of positive and negative interactions they had with particular trading partners. Because our chief concern here is to understand how a subject’s *perception* about a trading partner and their shared relationship affects the amount of trust and reciprocity she exhibits to the said trading partner, we do not view these subject errors to be an issue. While we do not report the analysis in this study, the overall significance of our results becomes even stronger if we strictly use computer records, not subject-reported records.

### Limitations

Of course, our chosen empirical strategy to study the process by which relationships based on trust and reciprocity develop in market settings raises issues of external validity. This concern is one that might be raised for every laboratory experiment. In recent years, one of the most prominent field experimentalists in economics, John A. List, and his co-author, Steve Levitt, questioned what economists may learn from laboratory experiments. At the center of their critique lied a concern about extrapolation. “[T]he critical assumption underlying the interpretation of data from lab experiments is that the insights gained can be extrapolated to the world beyond,” Levitt and List [134, p. 153] stated, but there are “many reasons to suspect that these laboratory findings might fail to generalize to real markets” [135, p. 909]. Levitt and List [134] then pointed to five factors that affect subject behavior and thus the degree to which we could extrapolate from the laboratory results: (1) the presence of moral and ethical considerations; (2) the nature and extent of scrutiny of one's actions by others; (3) the context in which the decision is embedded; (4) self-selection of the individuals making the decisions; and (5) the stakes of the game.

In a response to their criticisms, Camerer [136] argued that Levitt and List [134] exaggerated the difference in behavior that results from the margins on which laboratory experiments differ from field experiments. More provocatively, Camerer argued that external validity is irrelevant for a large class of laboratory experiments; it is necessary for studies whose principle aim is to inform policy, he said, but it is not necessary for studies whose principle aim is to understand general principles. Besides, he added, if external validity was a central concern, laboratory studies could be altered to better mirror the external environment of interest. Kessler and Vesterlund [137] seemed to agree with Camerer and argued that it is only relevant to ask whether the comparative statics, not the quantitative results (e.g. the actual magnitudes of measured treatment effect), are externally valid for most laboratory experiments. In addition, Fréchette [138] compared experimental studies that utilized student populations and that utilized people working in the industry (i.e. professionals) where a particular economic game was thought to be relevant. He found that results from the two subject pools tended to conform to the same comparative static predictions of an economic theory and tended to yield similar conclusions. (For more on this laboratory experiment-field experiment debate, see also [139–142]).

With regard to the criticisms against laboratory experiments, there is no way for one laboratory experiment to overcome challenges put forth by Levitt and List [134]. However, we specifically designed our market game to allow for the development of relationships characterized by trust and reciprocity, whilst allowing for the personalization/anonymity and the ability to defect/cheat that we often observe in real-world markets. When designing the two treatments in our experiment, we had in mind the farmers market (Market PE) and the financial market (Market IE). Since it is hard to isolate the exchange mechanism from other factors such as face-to-face interactions and communication in real-world farmers markets, the laboratory is the ideal place for us to examine whether market interactions alone (specifically the exchange process in isolation) can affect trusting and reciprocating behavior of former trading partners. For these reasons, we believe our experiment is uniquely positioned to not only inform us about the varying capacities of different market institutions to develop positive and negative relationships, but also to (indirectly) address a chief concern in social sciences about the social and moral consequences of market societies.

Another potential limitation of this experiment is the use of college students as subjects. As an anonymous reviewer appropriately remarked, “student responses can be useful when the activities touch upon things with which they have direct experience,” but they may be less useful when exploring “decisions on how to respond as buyers or sellers in an implied industrial market space.” Because many laboratory-based economic experiments utilize students, this is a concern about this substantial body of work, not just our experiment. That said, there are several possible responses. First, it would be incorrect to suggest that the students in the study had no experience buying and selling or forming bonds of trust and reciprocity prior to experiment. We cannot deny that students do generally have less experience in market settings than professionals (by whom we mean here individuals with considerable experience in markets as buyers and sellers). But if our arguments about the potential of market interactions generating trust are correct, then evidence of this (i.e. the observed treatment effect) should be even less apparent in our experiment with students as subjects than it would be had we used professionals as subjects. In this way, the fact that our results support our hypothesis despite the use of students as subjects should be viewed as bolstering rather than weakening our findings. Second, experimental economic studies that have compared the behavior of students and professionals as subjects (in terms of magnitude) have indeed found that students and professionals do behave differently (for example, Alevy et al. [143] and Abbink and Rockenbach [144]) although they can also behave similarly (for instance, Fehr and List [122] and Cooper et al. [145]). Since we are merely examining the *possibility* that market interactions can affect trust and reciprocity, and not trying to determine particular magnitudes or hoping to advance policy recommendations, we do not believe that the differences between students and professionals are likely to be relevant. Third, the laboratory experiments that Camerer [136] discussed in his study on the generalizability of lab results to the field generally relied on student subjects. His conclusion that results from most laboratory experiments do generalize into the field is also relevant and applicable here. For these reasons, we believe that our use of students as subjects is acceptable in this experiment.

## Hypotheses

Before presenting our results, we introduce the hypotheses that will be used to test our contention that positive and negative market interactions under certain market institutions can affect the trusting and reciprocating behavior of former trading partners.

Countless studies within experimental economics reported on how people behave differently towards others under varying degrees of anonymity and at varying social distances. Taken together, these studies demonstrated that subjects behaved more selfishly (or more immorally in general) when they could conceal their actions and when they felt less connected to their partners/counterparts. For instance, Hoffman et al. [146] reported that over 60% of the offers in a dictator game were $0 under the condition of complete anonymity in their strict double-blind treatment but that a bit over 40% of the offers were $0 when the condition of complete anonymity was relaxed in their single-blind treatments. Bohnet and Frey [147] observed how the proportion of equal splits in the dictator game increased as the social distance between dictators and their respective recipients decreased. They posited that a person’s motivation to behave fairly must (and can only) be intrinsic under conditions of anonymity and that the fairness norm must be (partially) activated when people can identify one another. Castillo and Leo [148] compared Player 2 behavior in a standard trust game to Player 2 behavior in a modified trust game where Player 2s made transfer decisions 80% of the time and were forced to keep all that was transferred to them 20% of the time. As such, Player 1s had difficulty deciphering Player 2s intentions when E$0 was returned in back transfers. They found that Player 2s in the modified trust game were more likely to exhibit selfish behavior and concluded that “[t]he fact that responders can hide selfish acts generates more selfish behavior” [148, p. 271].

Perhaps, the literature that most closely speaks to our topic of interest here is subject rematching in repeated games [149–154]. For instance, Andreoni [155], a seminal paper in this literature, saw that subjects who played repeated one-shot public goods games with different group members in each round (i.e. the stranger treatment) contributed significantly more than those who played the game repeatedly with the same group members (i.e. the partner treatment). He explained that the provision decay he observed in the partner treatment may arise due to subjects learning about the incentives of the game and thus discovering the dominant strategy through repeated play; since subjects learn at different paces, average provision gradually approaches zero. While we have no disagreement with his interpretation, we would also add that, as part of this learning process, his subjects likely learned that their partners were more or less free riders. Our contention that people can learn about the characters of their partners with whom they repeatedly interact and then incorporate this acquired information to their subsequent actions does not seem inconsistent with Andreoni’s findings.

Given the experimental evidence in the literatures cited above, we expect that people in a market typified by impersonal market exchanges will defect more than those in a market typified by personal market exchanges. In other words, we expect Market IE to have a higher defection rate than Market PE.

**Hypothesis 1 *(Defection rates)***. The defection rate in Market IE is higher than the defection rate in Market PE.

As the idiom goes, actions speak louder than words. It seems natural to presuppose that people do not trust those who have cheated them in the past and that they punish betrayals and reward trustworthiness. Fehr et al. [156], Berg et al. [83] and McCabe et al. [157], among others, observed how positive reciprocity occurs in trust games and gift exchange games. As such, if market interactions are affecting the trusting and reciprocating behavior of former trading partners, we expect people in Market PE to exhibit higher levels of trust and reciprocity towards those with whom they share positive relationships compared to those with whom they share negative relationships. We do not, however, expect to see differences in the treatment of positive and negative relationships in Market IE. First, while subjects in Market PE learn something about the trustworthiness of their trading partners, subjects in Market IE lack this ability to discriminate between people with whom they had positive or negative interactions. Second, while subjects in Market PE could feel the sting of personal betrayal, subjects in Market IE are insulated from the sting of personal betrayal even when it occurs. It is likely that people do not experience betrayals deeply in environments typified by impersonal market exchanges and therefore do not punish harshly and reward graciously. Third, we expect there to be a great deal of negative interactions in both of our experimental markets (but especially in Market PE). As a result, it is hard to imagine subjects in Market IE punishing subjects who cheated them harshly, since almost everyone is cheating them and because they are not feeling the sting of betrayal. Thus, we expect to observe the same levels of trust and reciprocity to be exhibited towards negative and positive relationships in Market IE.

**Hypothesis 2 (*Trust and reciprocity)***. Differential levels of trust and reciprocity by relationship types are observable in Market PE but are not evident in Market IE.

**Hypothesis 2a *(Trust in Market PE)***. Player 1 transfers to negative relationships are smaller than transfers to positive relationships in Market PE.

**Hypothesis 2b *(Reciprocity in Market PE)***. Player 2 transfers to negative relationships are smaller than transfers to positive relationships in Market PE.

**Hypothesis 2c *(Trust in Market IE)***. Player 1 transfers to negative relationships are identical to transfers to positive relationships in Market IE.

**Hypothesis 2d *(Reciprocity in Market IE)***. Player 2 transfers to negative relationships are identical transfers to positive relationships in Market IE.

Bohnet et al. [89] reported experimental evidence on how people were less willing to take risks when the outcome depended on whether or not other people proved to be trustworthy (even when the associated probabilities and payoffs were identical) compared to when the outcome was due to pure chance; in other words, people appeared to have betrayal aversion. Betrayal aversion has significant economic consequences because it hinders trust and cooperation [79, 89, 158]. Therefore, if the personal nature of the exchanges in Market PE is, in fact, leading to differences in the trusting and reciprocating behavior of subjects in that treatment, then we should expect to see evidence of betrayal aversion in Market PE. As such, trust shown to negative relationships in Market PE should be lower than the trust shown to negative relationships in Market IE.

**Hypothesis 3 *(Betrayal aversion)***. Player 1 transfers to negative relationships in Market PE are smaller than transfers to negative relationships in Market IE.

We expect these hypotheses to be borne out by the results of our experiment. Note that these hypotheses are silent on how subjects treat those with whom they do not share a positive or negative trading relationship (i.e. no relationship). We also have no prediction as to whether or not trust and reciprocity will differ at the treatment level.

## Results

Again, a total of 132 subjects participated in our experiment. For Market PE, we have observations from seven sessions of eight subjects and one session of 12 subjects for a total of 68 subjects. For Market IE, we have observations from eight sessions of eight subjects for a total of 64 subjects. Each session with eight subjects created 16 possible buyer-seller relationships and the one Market PE session with twelve subjects created a total of 36 possible buyer-seller relationships. Thus, we have observations on a total of 276 market relationships in our data: 148 buyer-seller relationships in Market PE and 128 buyer-seller relationships in Market IE. A total of 246 agreements were reached in Market PE and a total of 432 agreements were reached in Market IE. Following the convention in experimental economics, Player 2 transfers have been converted from tokens to proportions of the respective Player 1’s tripled transfer (i.e. $\frac{y}{3x}*100$). Unless otherwise stated, we report statistical results from two-sided Mann-Whitney tests.

**Fig 1:**
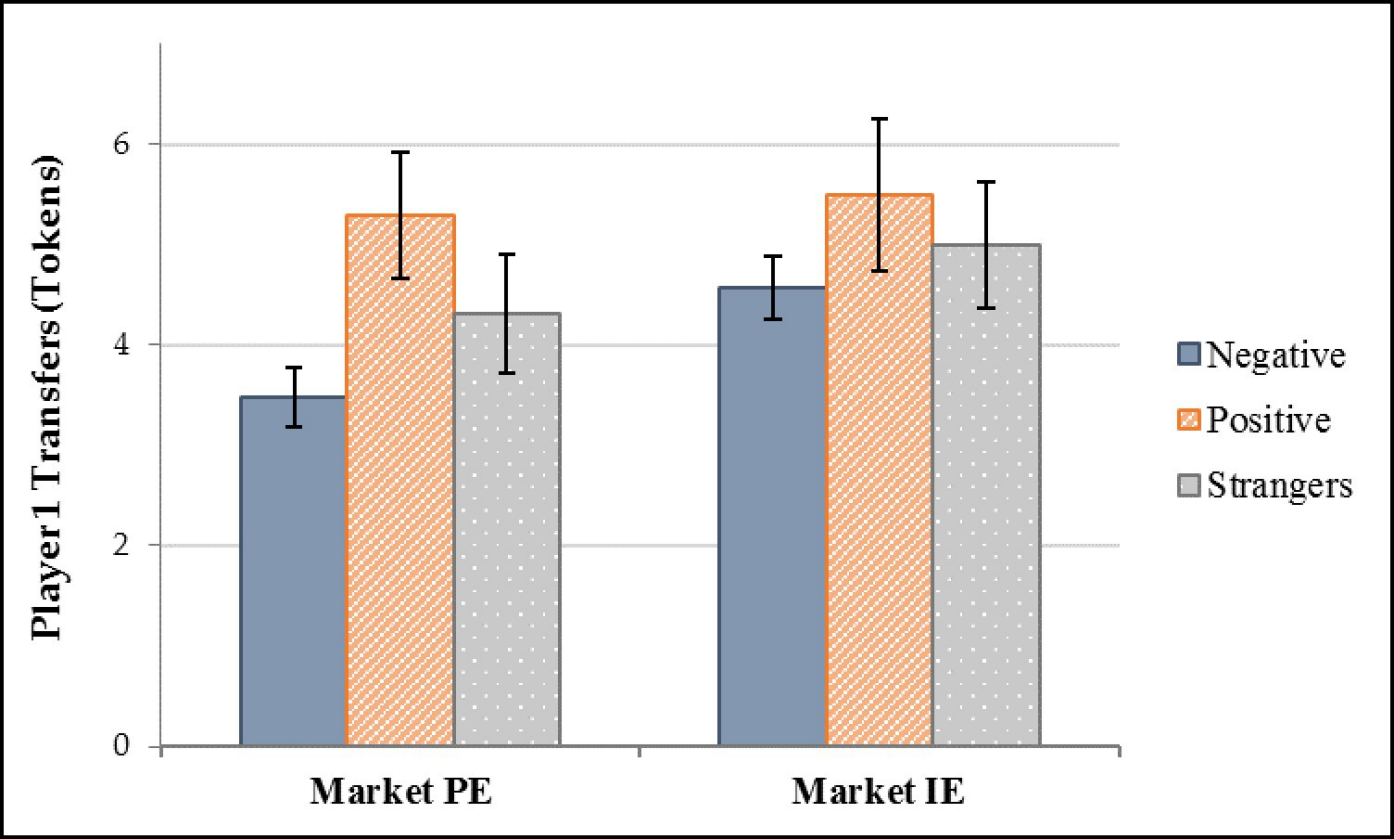
Player 1 transfers in Markets PE and IE by relationship typ. The error bars represent standard errors. Graph presents Player 1 transfers by different relationship types across treatments. The first set of bars represents Player 1 transfers in Market PE and the second set of bars represents Player 1 transfers in Market IE. Within each set of bars, from left to right, the bars represent negative relationships, positive relationships, and subject pairs with no trading relationships.

**Fig 2:**
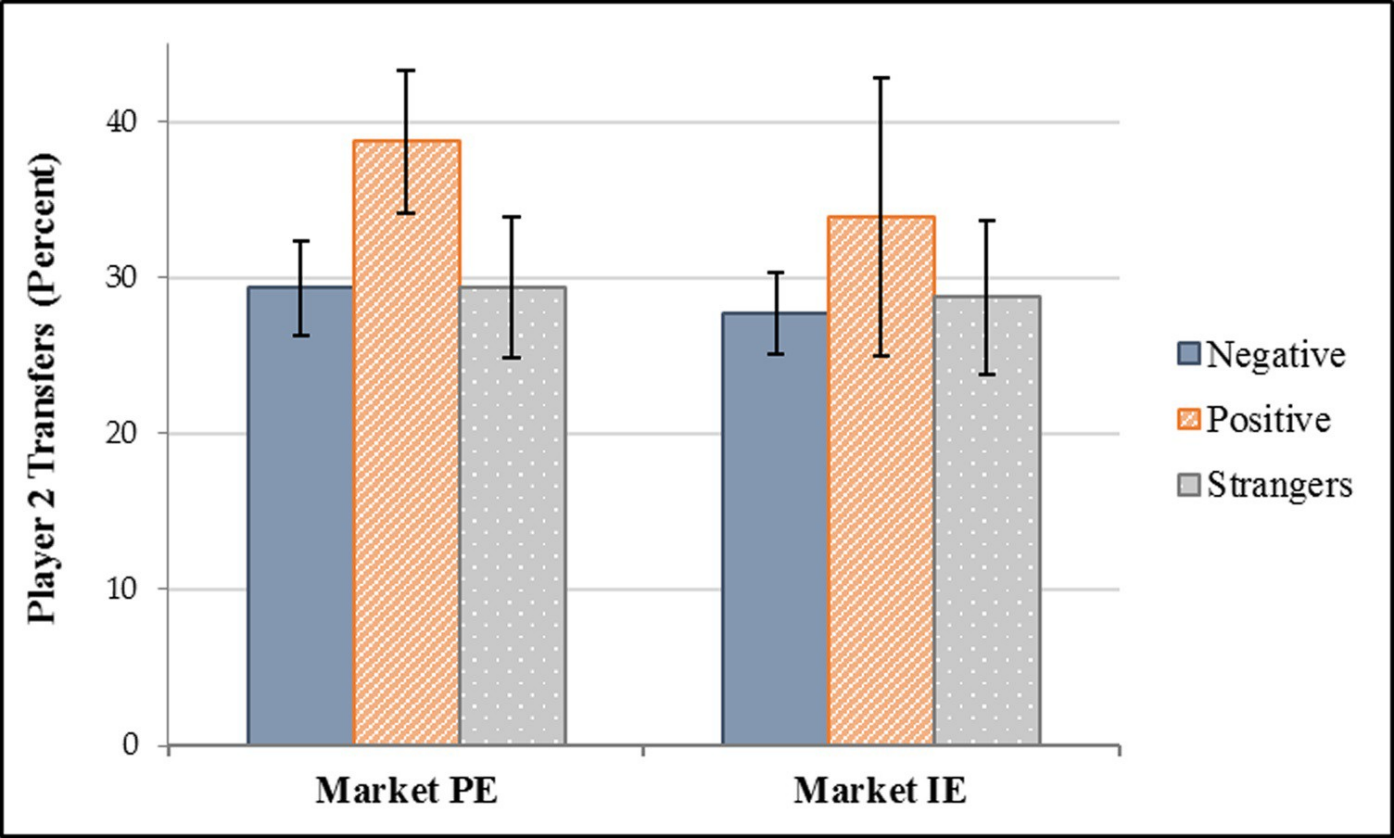
Player 1 transfers in Markets PE and IE by relationship type. The error bars represent standard errors. Graph presents Player 2 transfers by different relationship types across treatments. The first set of bars represents Player 2 transfers in Market PE and the second set of bars represents Player 2 transfers in Market IE. Within each set of bars, from left to right, the bars represent negative relationships, positive relationships, and subject pairs with no trading relationships.

Tables 1 and 2 present our summary statistics on trust and reciprocity by relationship type (see also Figs 1 and 2). Subjects exhibited significantly less trust in Market PE compared to Market IE (4.02 vs. 4.71, *p* = 0.043) and equally reciprocated in Market PE compared to Market IE (31.38% vs. 28.27%, *p* = 0.69) (Table 3).

**Table 1:** Summary of Player 1 transfers in Markets PE and IE by relationship type.

	Market PE		Market IE	
Treatment Average (Tokens)	4.02 (0.258) *N* = 148		4.71 (0.265) *N* = 128	
Relationship Type	Mean	n	Mean	n
**Negative**	3.48 (0.30)	84	4.57 (0.31)	98
**Positive**	5.29 (0.63)	28	5.5 (0.76)	10
**No**	4.31 (0.59)	36	5 (0.63)	20

Standard errors are reported in parentheses.

**Table 2:** Summary of Player 2 transfers in Markets PE and IE by relationship type.

	Market PE		Market IE	
Treatment Average (% Returned)	31.38 (2.377) *N* = 148		28.27 (2.216) *N* = 128	
Relationship Type	Mean	n^a^	Mean	n^a^
**Negative**	29.34 (3.00)	85	27.75 (2.59)	99
**Positive**	38.76 (4.56)	32	33.96 (8.94)	7
**No**	29.37 (4.55)	31	28.78 (4.92)	22

Standard errors are reported in parentheses. Percentage returned by Player 2 is calculated as Player 2’s token transfer divided by the respective Player 1’s tripled token transfer.

^a^ The sample sizes for Players 1 and 2 across relationship types are not identical due to subject errors in the record sheets. (See the “Defining seller-buyer relationships” subsection in the “Experimental design and procedure” section.)

**Table 3:** Comparisons of trust game transfers and defection rates across treatments.

		Z stat	p-value
**(1)**	**Player 1 Transfers**	2.026	0.043
**(2)**	**Player 2 Transfers**	-0.399	0.69
**(3)**	**Defection Rates**	-6.817	0.001
**(4)**	**Player 1 Transfers to Negative Relationships**	2.407	0.016

Table reports results from two-sided Mann-Whitney tests for the trust game transfers and from a two-sided proportions test for defection rates. Row (1) compares Player 1 transfers between Markets PE and IE. Row (2) compares Player 2 transfers between Markets PE and IE. Row (3) compares defection rates between Markets PE and IE. Row (4) compares Player 1 transfers to negative relationships between Markets PE and IE.

Result 1 *(Defection rates)*. The defection rate in Market PE is lower than the defection rate in Market IE.

### Support for result 1

Of the 246 agreements that were reached in Market PE, Proposers decided to defect on 58.1% of them. On the other hand, of the 432 agreements that were reached in Market IE, Proposers decided to defect on 82.2%. The defection rates were significantly different from one another (two-sided proportions test, *p* < 0.001) (Table 3).

**Result 2 *(Trust and reciprocity)***. Differential levels of trust and reciprocity by relationship types are observable in Market PE but are not evident in Market IE.

**Result 2a *(Trust in Market PE)***. Player 1 transfers to negative relationships are smaller than transfers to positive relationships in Market PE.

**Result 2b *(Reciprocity in Market PE)***. Player 2 transfers to negative relationships are smaller than transfers to positive relationships in Market PE.

**Result 2c *(Trust in Market IE)***. Player 1 transfers to negative relationships are statistically no different than transfers to positive relationships in Market IE.

**Result 2d *(Reciprocity in Market IE)***. Player 2 transfers to negative relationships are statistically no different transfers to positive relationships in Market IE.

### Support for result 2

Table 4 presents results from pairwise comparisons of Player 1 and Player 2 transfers to negative and positive relationships by treatment. In Market PE, Player 1 transfers to negative relationships were less than those to positive relationships (3.48 vs. 5.29, *p* = 0.012) and Player 2 transfers shown to negative relationships were less than those transfers to positive relationships (29.34% vs. 38.76%, *p* = 0.05). However, we found no evidence that trust and reciprocity shown to negative and positive relationships were different in Market IE; Player 1 transfers to negative relationships were statistically the same as those to positive relationships (4.57 vs. 5.5, *p* = 0.317); and Player 2 transfers to negative relationships were statistically no different to those to positive relationships (27.75% vs. 33.96%, *p* = 0.338).

**Table 4:** Comparison of trust game transfers to negative and positive relationships by treatment.

	(1) Market PE		(2) Market IE	
	Z stat	p-value	Z stat	p-value
**Player 1 Transfers**	-2.519	0.012	-1.001	0.317
**Player 2 Transfers**	-1.96	0.05	-0.957	0.338

Table presents results from two-sided Mann-Whitney tests. Column (1) compares trust game transfers to negative relationships and positive relationships in Market PE. Column (2) compares trust game transfers to negative relationships and positive relationships in Market IE.

Note that our claim here is about how people behave (with respect to Player 1 and Player 2 transfers) in different relationship types within treatment, not across treatments. Result 2 makes no claims about how Player 1 and Player 2 transfers compare across treatments keeping the relationship type constant.

When we compared Player 1 and Player 2 transfers across treatments for the same relationship type, we found that only Player 1 transfers to negative relationships are statistically different. More specifically, for positive relationships, Player 1 transfers in Market PE were statistically no different to those in Market IE (5.29 vs 5.5, *p* = 0.664) and Player 2 transfers in Market PE were statistically no different from those in Market IE (38.76% vs. 33.96%, *p* = 0.755). For negative relationships, Player 1 transfers in Market PE were smaller than those in Market IE (3.48 vs 4.57, *p* = 0.016) and Player 2 transfers in Market PE were statistically no different from those in Market IE (29.34% vs. 27.75%, *p* = 0.832).

**Result 3 *(Betrayal aversion)***. Player 1 transfers to negative relationships in Market PE are smaller than transfers to negative relationships in Market IE.

### Support for result 3

We observe betrayal aversion in our experiment. Player 1 transfers to negative relationships in Market PE is significantly smaller than Player 1 transfers to negative relationships in Market IE (3.48 vs. 4.57, *Z* = − 2.407, *p* = 0.016) (Table 3).

## Discussion and conclusion

The potential of markets (or market exchanges) to facilitate or discourage the emergence of trust and the social relationships that depend on trust is relatively understudied. There are, however, a few exceptions in experimental economics. Crockett et al. [159, p. 1163] conducted an experiment in which,

. . . individuals are privately informed of their home production and consumption opportunities, must discover their potential to gain through specialisation–provided that they find and develop bilateral or multilateral trading relationships with others having complementary circumstances–and must rely on trust and repeated interaction to enforce agreements and develop their own institutional relations.

They concluded that three stages of learning are necessary to achieve competitive equilibrium, which are [160, p. 281–282]:

. . . (1) discovering the ability to exchange, which may require “mind-reading” (inferring intentions from words and actions) and imitation; (2) finding a suitably endowed trading partner with whom one can benefit from exchange through specialization; and (3) building the relationship by increasing specialization other time.

Building on Crockett et al.’s [159] study, Kimbrough et al. [160] investigated how and when impersonal market exchange and long-distance trade bore out of local specialization. They described, among a number of other findings, how conversations among the members of the same group were “personal and casual” [160, p. 297] and demonstrated in-group/out-group sentiments. Similarly, Kimbrough et al. [161] explored the institutional conditions under which impersonal market exchanges emerge from personal social exchanges. They [161, p. 1009] found that “a history of unenforced property rights hinders [their] subjects’ ability to develop the requisite personal social arrangements to support specialization and effectively exploit impersonal long-distance trade.” While Crockett et al. [159] and Kimbrough et al. [160–161] addressed a question dissimilar from our own here (i.e. how, broadly speaking, markets grow out of personal exchanges), their studies also substantiate how strong bilateral relationships can emerge in economic exchange settings and how such relationships are necessary to achieve immense wealth.

In our study, we used a laboratory experiment to study whether positive and negative market interactions can affect the trusting and reciprocating behavior of former trading partners and whether the personal/impersonal nature of market exchanges can influence the levels of trust and reciprocity that they exhibit. We found evidence that suggests that positive and negative market interactions can affect the trusting and reciprocating behavior of former trading partners. But we found that past market dealings only affected the trusting and reciprocating behavior of subjects who participated in our experimental market where exchanges were more personal but did not affect trust and reciprocity between trading partners who participated in our experimental market where exchanges were more impersonal. In Market PE, people exhibited higher levels of trust in trading partners with whom they shared positive relationships than to those with whom they shared negative relationships. In Market IE, people trust those with whom they shared positive and negative relationships more or less equally. In short, our results speak to how different market institutions can affect how sensitive trading partners’ trusting and reciprocating behavior is to their previous dealings.

One possible reason why subjects in Market PE, but not those in Market IE, trust and reciprocate to negative and positive relationships quite distinctly may be that certain types of markets enable market participants to learn about one another by the way they go about buying and selling goods and services [19–21]. The market process, Hayek explained, allows market participants to learn about the profitability of different opportunities and courses of action, and to adjust their expectations and plans accordingly. While Hayek [21, p. 109] likely meant discovering who most cheaply produces a particular good when he wrote about discovering “who will serve us well,” his argument does not preclude learning which trading partners would best boost our long-term profits. And, of course, information about our trading partner’s trustworthiness and tendency to follow through on agreements would be helpful as we determine our expected long-term profitability. As Hayek [21, p. 109] described,

In actual life, the fact that our inadequate knowledge of the available commodities or services is made up for by our experience with the persons or firms supplying them–that competition is in a large measure competition for reputation or good will–is one of the most important facts which enables us to solve our daily problems. The function of competition is here precisely to teach us *who* will serve us well: which grocer or travel agency, which department store or hotel, which doctor or solicitor, we can expect to provide the most satisfactory solution for whatever particular personal problem we may have to face.

It is also worth noting that, if Market IE also enabled market participants to learn about one another like Market PE, then we should have observed much less trust in Market IE than Market PE because the defection rate in Market IE was over 40% higher than that in Market PE. Trust in Market IE was, in fact, higher than trust in Market PE (4.71 vs. 4.02, *p* = 0.043), suggesting the learning process we describe here did not occur in Market IE.

Our result on betrayal aversion is consistent with findings by Bohnet et al. [90] with one subtle difference: they demonstrated the existence of betrayal aversion towards *unknown* others and we demonstrated the existence of betrayal aversion towards *known* others. According to Bohnet et al. [90], people choose to act cautiously because they want to minimize the total cost associated with their decisions, not knowing precisely with whom they may ultimately interact. In our experiment, in particular Market PE, a negative (and personal) relationship with a trading partner may develop in three ways: the trading partner tended to violate the subject’s trust in the market game; the subject tended to violate the trading partner’s trust in the market game; or both the trading partner and the subject tended to violate each other’s trust in the market game. Over the course of our market game, our subjects likely have learned which trading partners would betray their trust. Having learned that, it is unsurprising that our subjects would subsequently decide to display significantly less trust towards trading partners with whom they shared negative relationships. In other words, our experiment suggests that people do act rationally and do minimize total cost associated with their decisions, including emotional costs, by choosing not to trust people who have proven to be untrustworthy.

Finally, our results here suggest that the same opportunity to learn about trading partners (i.e. execute/defect decisions) does not work equally well across all market contexts. For instance, in a bilateral, simultaneous offer environment (like Market PE here), Choi and Storr [23] established how the ability to make execute/defect decisions served as an opportunity to “really get to know” their partners and learn about their partners’ likelihood to follow through on agreements. Without the execute/defect decision, subjects showed no difference in the way they trusted and reciprocated towards negative and positive relationships even in the same bilateral, simultaneous offer environment. Our results here suggest that to the extent that the execute/defect decision serves as a learning opportunity, it is only meaningful information in markets characterized by personal exchange.

## Supporting information

S1 Data (XLSX)

S2 Data (PDF)

S3 Data (PDF)

S4 Data (PDF)

S1 File (DOCX)
