# Supplementary material for: Market interactions, trust and reciprocity
Source: PLoS One. 2020 May 7;15(5):e0232704. doi: 10.1371/journal.pone.0232704 (PMC7205303; doi:10.1371/journal.pone.0232704)
Supplement: S3 Data — (PDF) [file pone.0232704.s003.pdf]

Instructions for Market PE

WELCOME!

Please turn off all electronic devices if you have not done so and place them in your bags or under your desk. Throughout the experiment, please do not talk to anybody else and there must be absolute silence. If you have any questions, please raise your hand and the experimenter will come to personally assist you.

Thank you for participating in this experiment. By showing up on time, you have automatically earned a $5 on-time payment. At the end of the study, you will be paid privately in cash.

Experimental Name (i.e. Alias)

Today, you will be randomly assigned an alias by the computer at the beginning of the experiment. You will keep the same alias throughout today's experiment. Please do not share this alias with other participants. Your real name will never be revealed to anyone or recorded in this study and will only be known by your alias throughout this study.

The Experiment

You will perform two tasks. These instructions will describe your First Task. Instructions for the Second Task will be provided to you after the First Task is finished.

The experimenter will provide you with a record sheet before the start of the first paid round of each task. **<u>After each round ends, make sure to fill out each column with the appropriate information.</u>** These record sheets will be used during your final cash payment process at the end of the experiment, so please make sure to write legibly.

First Task

The Game

You will play a market game. There are two roles in this game – buyer and seller. You will play this game for 11 trading rounds (1 practice round and 10 paid rounds) and each trading round lasts 2.5 minutes. The market consists of 4 buyers and 4 sellers and you will be randomly assigned to one of these roles at the beginning of the first paid round. Your role will never change throughout this task.

Flow Chart: Overview of what happens in each round

Stage 1
- Computer randomly gives a cash/cost value to each participant

Stage 2
- Buyers and sellers negotiate prices by making offers to each other

Stage 3
- If you successfully traded:
  Offer creators: Decides whether to execute or disregard the trade
  Offer recipients: Views what they would earn if offer creator executes and disregards the trade

Stage 4
- Displays relevant information for your record sheet

At the beginning of each round, each buyer will be given some cash (in experimental dollars) and each seller will be given one unit of a good and a production cost. The buyer's objective is to purchase a unit of good from a seller at a price less than or equal to his cash. The seller's objective is to sell his good to a buyer at a price greater than or equal to his cost. This is achieved by negotiating: buyers can only make offers to sellers (e.g. "I would like to buy your good at the price of E$54.") and sellers can only make offers to buyers (e.g. "I would like to sell my good at the price of E$54.") You can negotiate as many times with whomever you wish within a round. The only restrictions are (1) you can have at most one successful trade at the end of each round and (2) you can only have one open offer at a time. The next section will describe how you can accept, reject or withdraw offers.

Note that cash and cost values are your private information and no one will ever know your value. You will receive a new number at the beginning of each round. The computer will randomly choose a number between E$10 and E$100 and each value has an equal chance of being chosen.

In each trading round, you earn positive round profits by successfully trading with someone. When a buyer successfully purchases a good, his round profits is calculated as his cash minus the negotiated price. When a seller successfully sells his good, his round profits is calculated as the negotiated price minus his cost. If you do not successfully make a trade during a round, your round profits will be zero. But there is one other way with which you may earn zero profits.

Once a trade has successfully occurred, the person who sent the offer always has a choice to execute or disregard this trade. For example, suppose you are a seller and a buyer has accepted your offer. If you decide to execute this trade, both you and your trading partner will earn round profits as described in the

above paragraph. But if you decide to disregard this trade, your round profit will be the negotiated price plus E$10 while your partner's round profit will be zero. Similarly, suppose you are a buyer and a seller has accepted your offer. If you decide to disregard the trade, your round profit will be your cash plus E$10 while your partner's round profit will be zero. (Again, both you and your partner will earn round profits as described above if you decide to execute this trade.)

The round ends when the computer displays your partner's name, his execute/disregard decision (if applicable) and your round profit. A new trading round will begin with everyone receiving a new cash or cost value. Any successful trades in previous trading rounds will not affect your subsequent round profits.

Example:

Consider a case where a buyer is endowed with E$75 and a seller's cost value is E$10. Suppose they successfully completed a trade and their negotiated trade price is E$54.

- If the trade is executed, the buyer's round profit is $E\$75 - E\$54 = \boxed{E\$21}$
  the seller's round profit is $E\$54 - E\$10 = \boxed{E\$44}$

- Suppose the buyer made the offer and decided to disregard the trade.
  The buyer's round profit is $E\$75 + E\$10 = \boxed{E\$85}$
  The seller's round profit is $\boxed{E\$0}$

- Suppose the seller made the offer and decided to disregard the trade.
  The seller's round profit is $E\$54 + E\$10 = \boxed{E\$64}$
  The buyer's round profit is $\boxed{E\$0}$

- If this buyer and seller do not successfully trade with anyone, their round profits will be E$0.

Cash Payment from Second Task

At the end of the experiment, one round will be randomly chosen to determine your cash payment from the First Task. Every 5 experimental dollars from this task will be worth 2 US dollars.

Do not talk or indicate in any way to your fellow participants that may reveal your experimental identity.

How to Play the Game

1. How to make offers using Offer Creation box

As a buyer, you can only negotiate with sellers and likewise for sellers. To send an offer, enter a number next to "Your Offer" and select a specific participant as the recipient. (In the example on the right, the possible recipients are A, B, C and D.) When you are sure with your offer, click "Submit Offer."

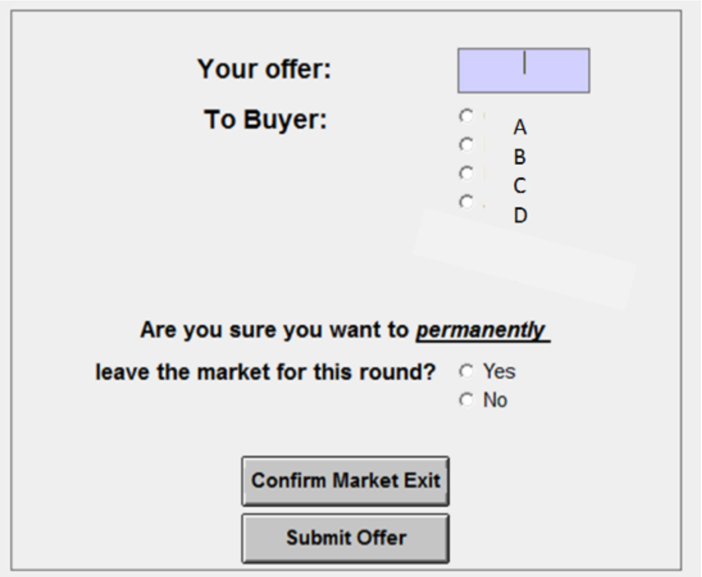

Remember your objective is to make a positive profit. As a buyer, your offer cannot be higher than the cash you hold. As a seller, your offer cannot be lower than your cost. Besides these restrictions, you are allowed to make any offers.

<u>However, you can only send one offer at a time.</u> This means that any offer you send must first be either accepted or rejected by the recipient or withdrawn (i.e. cancelled by yourself) before you can send another offer.

2. How to identify offers using "Your Open Offers" Table

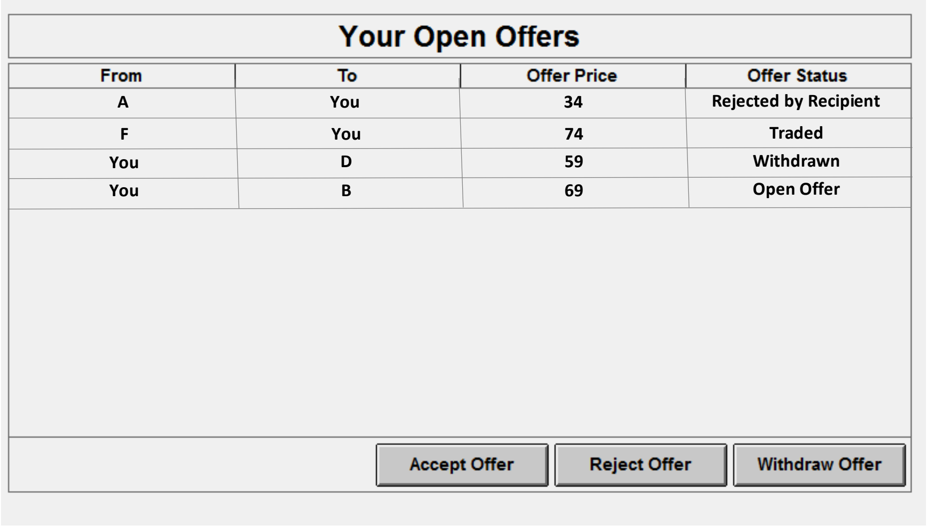

Your Open Offers

From	To	Offer Price	Offer Status
A	You	34	Rejected by Recipient
F	You	74	Traded
You	D	59	Withdrawn
You	B	69	Open Offer

Accept Offer Reject Offer Withdraw Offer

Whenever you make and receive offers, these offers will appear in your open offers table. No one else in the room can ever see your offers. The "From" column displays the creators of offers (i.e. the person who sent the offer) and the "To" column displays the recipients of the offers. The "Offer Price" column displays the price proposed by the creators of the offers. For example, the first row of the table above shows an offer sent from Participant A to you for a proposed price of E$34.

You can check the status of the offers by looking at the rightmost column. There are 5 possible statuses:

Open Offer	If you sent this offer, it has not been accepted or rejected by the recipient.<br><br>If you received this offer, you have not yet accepted or rejected this offer.
Rejected by Recipient	This offer has been rejected by the recipient.
Traded	This offer has been accepted and a trade has successfully occurred.
Withdrawn	The creator of this offer has withdrawn the offer.
Offer Expired	The creator or recipient of this offer has successfully traded with someone else for this round.

Returning to the "Your Open Offers" table above, the "Offer Status" column shows that you have rejected an offer of E$34 from Participant A and accepted an offer of E$74 from Participant F.

3. How to reject and accept offers sent to you

When you receive offers, you can reject or accept them using the buttons located at the bottom of "Your Open Offers" table.

To reject an offer, first click on the offer price you want to reject. (The row will highlight blue.) Then click the "Reject" button. The status of the offer will then change from "Open Offer" to "Rejected by recipient." You cannot reject your own offers.

Accepting an offer sent to you is a <u>**3 step process**</u>. ***You must follow these steps in order to make sure your trade is properly recorded by the computer.***

a. Click on the offer price you wish to accept. (The row will highlight blue.) Then click the "Accept" button. The status of the offer will change from "Open Offer" to "Traded."
b. Go to the Offer Creation box. Answer "Are you sure you want to <u>*permanently*</u> leave the market for this round?" with a "Yes" and click the "Confirm Market Exit" button.
c. Click the red "Finished" button at the bottom of your screen.

<u>***You can only accept one offer per round. In other words, you can successfully trade once per round.***</u>

4. How to identify who has left the market

At some point, you may want to find out who has left the market either because you have been waiting on a recipient to respond (i.e. accept or reject) your offer or because you want to make a new offer to a new recipient. You can check who has left the market by looking at the "Buyers/Sellers who have left

the market" table such as the one seen below. During the task, buyers will only see sellers' aliases in this table and, similarly, sellers will only see buyers' aliases.

Buyers who have left the market

Buyer's Name	Market Status

5. Withdrawing offers you have sent

During the game, you may want to withdraw the offers you have sent. In order to do so, click on the relevant offer price and click the "Withdraw button."

Unfortunately, due to the limitations of the software, you must also withdraw the offer you have sent if its status changes to "Offer Expired" or "Rejected by Recipient" if you want to continue making offers. *This means that you must withdraw the offers you have sent first before you use the "Offer creation" box to make another offer.*

6. As an offer creator, how to execute or disregard a trade

Once the negotiation stage is over, the computer will move you onto a new decision screen if you were able to successfully trade. On this screen, offer creators are asked whether or not they wish to execute the trade and the computer lists the profits you could earn if you execute the trade and if you disregard the trade. More specifically, the computer will give a seller an "opportunity to decide whether or not you will deliver the good after receiving payment" and will give a buyer an "opportunity to decide whether or not you will pay your partner after he delivers the good."

Answer the question, "Would you like to deliver the good after you receive payment?" / "Would you like to pay your partner after you receive the good?" with a "Yes" if you wish to execute (i.e. go through with) the trade and with a "No" if you wish to disregard (i.e. not go through with) the trade. After you are confident with your answer, you must click the "Continue" button at the bottom of the screen.

Offer recipients will see what they could earn if their partner executes the trade and if he disregards the trade, but will not be asked to make the execute/disregard decision.

If you were not able to successfully trade with no one, the computer will simply show you a screen that states you did not trade with anyone.

Second Task

The Game

There are two roles of players in this game – Player 1 and Player 2. You will each be randomly assigned to one of these two roles and will then play this game with 4 players in the other role. Every player will be endowed with 10 tokens at the beginning of the game for each paired partner.

First, Player 1 will be given the opportunity to transfer some, all or none of his 10 tokens to Player 2.

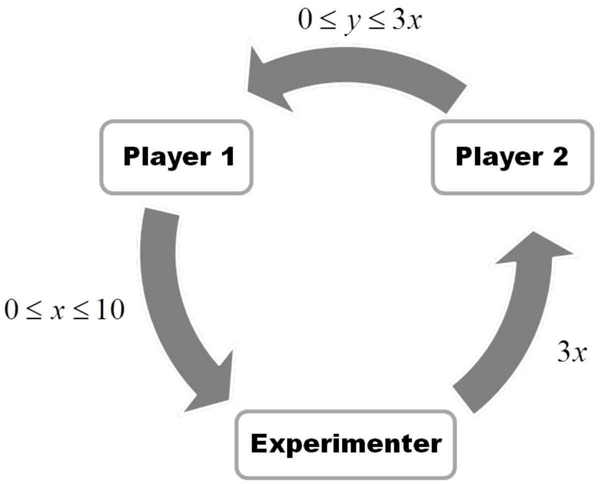

(Player 1’s transfer is represented by x in the diagram.) Then, the experimenter will multiply x by 3 before it is sent to Player 2.

Second, Player 2 will observe Player 1’s tripled transfer and then have an opportunity to transfer some, all or none of this amount back to Player 1. (Player 2’s transfer is represented by y in the diagram.) To minimize confusion, the computer will only display the tripled transfer on Player 2’s decision screen.

The game then ends once Player 2 completes her transfer.

As Player 1, each of your transfers must be between and including 0 and 10 tokens. As Player 2, each of your transfers must be less than or equal to the tripled transfer sent by your respective Player 1.

Your Token Earnings

For each of your partners, your earnings will be calculated by the computer and will be privately displayed on your screen. For your information, your earnings will be calculated in the following way.

Player 1’s round earnings are the endowment of 10 tokens minus the number of tokens sent to Player 2 plus the number of tokens sent back by Player 2. (Alternatively, it is expressed as $10 - x + y$.)

Player 2’s round earnings are his endowment of 10 tokens plus the number of tokens sent by Player 1 minus the number of tokens sent back to Player 1. (Alternatively, it is expressed as $10 + 3x - y$.)

Example 1

Suppose Player 1 transfers 2 tokens to Player 2: $x = 2\ tokens$
Before this transfer reaches Player 2, it is tripled by the experimenter: $3x = 6\ tokens$
Then, Player 2 can send any amount between and including 0 and 6 tokens back to Player 1.

If Player 2 decides to send back 3 tokens, Player 1 will earn: $10 - 2 + 3 = \boxed{11\ tokens}$
Player 2 will earn: $10 + 6 - 3 = \boxed{13\ tokens}$

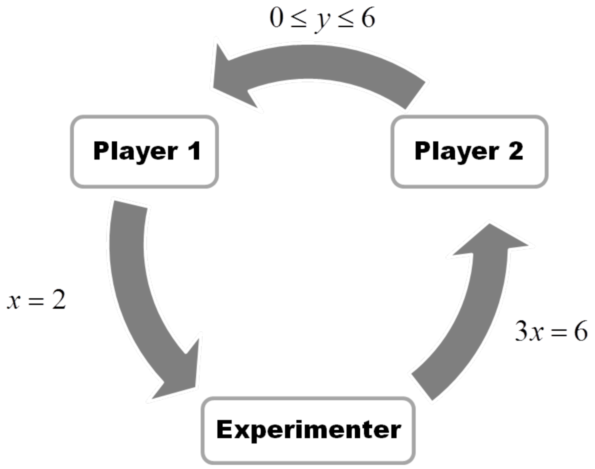

Example 2

Suppose Player 1 transfers 9 tokens to Player 2: $x = 9\ tokens$
Before this transfer reaches Player 2, it is tripled by the experimenter: $3x = 27\ tokens$
Then, Player 2 can send any amount between and including 0 and 27 tokens back to Player 1.

If Player 2 decides to send back 19 tokens, Player 1 will earn: $10 - 9 + 19 = \boxed{20\ tokens}$
Player 2 will earn: $10 + 27 - 19 = \boxed{18\ tokens}$

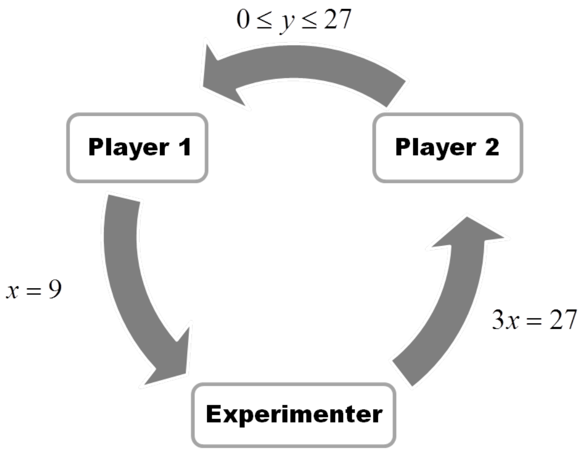

Details about this Game

- You will play this game once with each of your 4 partners. This means that you will be endowed with 10 tokens for each partner. If you are Player 1, each of your transfers must be between and including 0 and 10 tokens and may not exceed 10 tokens.
- All 4 games will all be played simultaneously.
- All of your interactions are private. This means that no one but you and your paired partner will ever know the results of your private interaction. (For example, if you are partnered with Justin and Andrew, only you and Justin will know the results of your private interaction. Likewise, only you and Andrew will know the results of your private interaction. Justin will never find out your decision for Andrew and vice versa. Similarly, you will never know the results of Justin and Andrew's private interaction.)
- ***There will be no practice rounds for this task.*** For your reference, sample screenshots will be provided for you after the experimenter finishes reading these instructions.
- *Again, remember to fill out your record sheet at the end of each round. This record sheet will be used during your final cash payment process at the end of the experiment.*

At the end of this Task

The computer will display some questions on your screen as part of the post-experiment survey. Please answer these questions to the best of your ability. The rest of the post-experiment survey will be provided to you by the experimenter after this task is finished.

Cash Payment from the Second Task

At the end of the experiment, a partner will be randomly chosen and you will be paid according to how much you earned while being paired to this partner. Your token earnings will be converted using an exchange rate of 1 US dollar to 3 tokens.

Do not talk or indicate in any way to your fellow participants that may reveal your experimental identity.
