# Supplementary material for: Market interactions, trust and reciprocity
Source: PLoS One. 2020 May 7;15(5):e0232704. doi: 10.1371/journal.pone.0232704 (PMC7205303; doi:10.1371/journal.pone.0232704)
Supplement: S4 Data — (PDF) [file pone.0232704.s004.pdf]

Instructions for Risk Attitude Measure in Post Experiment Survey

10. On the next page, you will see a list of 10 decisions. Each decision will be a paired choice between "Option A" and "Option B," just you see on the next page. Your task is to determine whether you prefer the lottery represented by Option A or the lottery represented by Option B for each decision row. But only one of them will be used in the end to determine your earnings. Before you start making your ten choices, please let me explain how these choices will affect your earnings for this part of the experiment. <u>Please note that you will only be paid for this part of the experiment if and only if you complete this survey.</u>

A 10-sided die that will be used to determine payoffs; the faces are numbered from 1 to 10 (the "0" face of the die will serve as 10.) After you have made all of your choices, we will throw this die twice in the room next door: once to select one of the ten decisions to be used and a second time to determine what your payoff is for the option you chose for the particular decision selected. Even though you will make 10 decisions, only one of these will end up affecting your earnings, but you will not know in advance which decision will be used. Obviously, each decision has an equal chance of being used in the end.

For example, please look at Decision Row 1 in the table on the next page. Option A will pay $2.00 with 10% chance and it will pay $1.60 with 90% chance. Option B will yield $3.85 with 10% chance and it will pay $0.10 with 90% chance. As another example, please look at Decision Row 2. Option A will pay $2.00 with 20% chance and it will pay $1.60 with 80% chance. Option B will yield $3.85 with 20% chance and it will pay $0.10 with 80% chance.

The other decisions are similar, except that as you move down the table, the chances of the higher payoff for each option increase. In fact, as you will see on the next page, Decision Row 10 will yield the highest payoff for sure, so your choice will be between $2.00 and $3.85.

For each decision row, you must choose between Option A and Option B. You may choose A for some decisions rows and B for other rows. Once you make a decision, you will make check marks in the middle of the table to represent your choices. Check the box on the left for A or the box on the right for B.

Please raise your hand if you have any questions.

Option A		Option B
**1/10** of $2.00, **9/10** of $1.60		**1/10** of $3.85, **9/10** of $0.10
**2/10** of $2.00, **8/10** of $1.60		**2/10** of $3.85, **8/10** of $0.10
**3/10** of $2.00, **7/10** of $1.60		**3/10** of $3.85, **7/10** of $0.10
**4/10** of $2.00, **6/10** of $1.60		**4/10** of $3.85, **6/10** of $0.10
**5/10** of $2.00, **5/10** of $1.60		**5/10** of $3.85, **5/10** of $0.10
**6/10** of $2.00, **4/10** of $1.60		**6/10** of $3.85, **4/10** of $0.10
**7/10** of $2.00, **3/10** of $1.60		**7/10** of $3.85, **3/10** of $0.10
**8/10** of $2.00, **2/10** of $1.60		**8/10** of $3.85, **2/10** of $0.10
**9/10** of $2.00, **1/10** of $1.60		**9/10** of $3.85, **1/10** of $0.10
**10/10** of $2.00, **0/10** of $1.60		**10/10** of $3.85, **0/10** of $0.10
