# Supplementary material for: Market interactions, trust and reciprocity
Source: PLoS One. 2020 May 7;15(5):e0232704. doi: 10.1371/journal.pone.0232704 (PMC7205303; doi:10.1371/journal.pone.0232704)
Supplement: S1 File — (DOCX) [file pone.0232704.s005.docx]

# **Supporting Information**

## **Alternative Definitions of Relationships**

It may seem intuitive to expect people’s decisions regarding how much trust and reciprocity to place in a known associate to depend on their overall experiences with their associates. However, overall experience may not be the only definer of relationships; first and last impressions could also predominate decisions regarding how much trust and reciprocity to place in someone. In this section, we evaluate our main results in two alternative ways through which subjects may assess the nature of their (market) relationships with each other: first impressions and last interactions.

There are experimental studies that speak to how first impressions can matter for economic decisions (e.g. Eckel and Petrie 2011; Centorrino et al. 2015). To the best of our knowledge, there is no experimental studies that directly address last interactions. Last interactions, as we define them here and elsewhere (Choi and Storr 2018), are distinct from last round (or end-of-game) effects. The closest analogy to our last impressions may be the restart effect (e.g. Andreoni 1988).

To evaluate the impact of first and last market interactions on trust and reciprocity, we classify each relationship between $buyer i$ and $seller j$ by whether their first or last agreement was executed or defected. If their first interaction ended positively, we classify such a relationship as a ***positive (trading) relationship by first impression*** (denoted as ${positive}_{first}$ in the results section for simplicity). If their first interaction ended negatively, we classify such a relationship as a ***negative (trading) relationship by first impression*** (denoted as ${negative}_{first}$). We parallel this convention and classify relationships with positive last interactions as ***positive (trading) relationships by last interaction*** (denoted as ${positive}_{last}$) and relationships with negative last interactions as ***negative (trading) relationships by last interaction*** (denoted as ${negative}_{last}$). Regardless of the way we measure and define relationships, a buyer and a seller who never once reached an agreement with each other across the ten trading rounds are defined as having ***no (trading) relationship***.

Tables S1 and S2 present our summary statistics on trust and reciprocity by relationship type defined by first impressions and last interactions.

Table S1: Summary of Player 1 transfers in Markets PE and IE by relationship type

|  | **Market PE** | | | | | | **Market IE** | | | |
| --- | --- | --- | --- | --- | --- | --- | --- | --- | --- | --- |
| **Treatment Average (Tokens)** | **4.02 (0.258)** $\boldsymbol{N=148}$ | | | | | **4.71 (0.265)** $\boldsymbol{N=128}$ | | | | |
| **Relationship Type** | **First Impression** | | **Last Interaction** | | | **First Impression** | | | **Last Interaction** | |
|  | **Mean** | **n** | **Mean** | **Mean** | | **Mean** | | **n** | **Mean** | **n** |
| **Negative** | 3.53  (0.30) | 68 | 3.56  (0.30) | | 89 | 5 (0.63) | | 20 | 4.57  (0.32) | 95 |
| **Positive** | 4.55  (0.67) | 44 | 5.35  (0.67) | | 23 | 5.5 (0.60) | | 22 | 5.23 (0.63) | 13 |
| **No** | 4.31  (0.59) | 36 | 4.31  (0.59) | | 36 | 4.44  (0.33) | | 86 | 5  (0.63) | 20 |

Standard errors are reported in parentheses.

Table S2: Summary of Player 2 transfers in Markets PE and IE by relationship type

|  | **Market PE** | | | | | **Market IE** | | | |
| --- | --- | --- | --- | --- | --- | --- | --- | --- | --- |
| **Treatment Average (% Returned)** | **31.38 (2.377)** $\boldsymbol{N=148}$ | | | | **28.27 (2.216)** $\boldsymbol{N=128}$ | | | | |
| **Relationship Type** | **First Impression** | | **Last Interaction** | | **First Impression** | | | **Last Interaction** | |
|  | **Mean** | **n**^a^ | **Mean** | **n**^a^ | **Mean** | | **n** | **Mean** | **n**^a^ |
| **Negative** | 29.21  (2.94) | 65 | 28.63  (2.94) | 86 | 28.78 (4.94) | | 22 | 27.98  (2.65) | 96 |
| **Positive** | 35.30  (4.66) | 52 | 41.04  (4.66) | 31 | 29.78 (5.88) | | 22 | 29.95  (7.19) | 10 |
| **No** | 29.37 (4.55) | 31 | 29.37 (4.55) | 31 | 29.74 (2.75) | | 84 | 28.78  (4.92) | 22 |

Standard errors are reported in parentheses. Percentage returned by Player 2 is calculated as Player 2’s token transfer divided by the respective Player 1’s tripled token transfer.

^a^ The sample sizes for Players 1 and 2 across relationship types are not identical due to subject errors in the record sheets. (See the “Defining seller-buyer relationships” subsection in the “Experimental design and procedure” section.)

**Result 2 *(Trust and reciprocity)*.** Differential levels of trust and reciprocity by relationship types are observable in Market PE but are not evident in Market IE.

**Result 2a *(Trust in Market PE)*.** Player 1 transfers to negative relationships are smaller than transfers to positive relationships in Market PE.

**Result 2b *(Reciprocity in Market PE)*.** Player 2 transfers to negative relationships are smaller than transfers to positive relationships in Market PE.

**Result 2c *(Trust in Market IE)*.** Player 1 transfers to negative relationships are statistically no different to transfers to positive relationships in Market IE.

**Result 2d *(Reciprocity in Market IE)*.** Player 2 transfers to negative relationships are statistically no different transfers to positive relationships in Market IE.

***Support for Result 2***. Table S3 presents results from pairwise comparisons of Player 1 and Player 2 transfers to negative and positive relationships defined as first impression and last interaction by treatment. In Market PE, Player 1 transfers to negative relationships were less than those to positive relationships no matter how we define relationships: transfers to ${negative}_{first}$ were statistically smaller than those to ${positive}_{first}$ (3.53 vs. 4.55, $p=0.093$); and transfers to ${negative}_{last}$ were statistically smaller than those to ${positive}_{last}$ (3.56 vs. 5.35, $p=0.017$). For Player 2s, only relationships defined by last interaction show statistical difference: transfers to ${negative}_{first}$ were smaller than those to ${positive}_{first}$ in magnitude, but were not statistically different from one another (29.21% vs. 35.30%, $p=0.155$); and transfers to ${negative}_{last}$ were statistically smaller than those to ${positive}_{last}$ (28.63% vs.41.04%, $p=0.017$).

While most pairwise comparisons for Market PE revealed significant differences in how people treat those with whom they share negative relationships compared to those with whom they share positive relationships, we did not observe any differences in Market IE: Player 1 transfers to ${negative}_{first}$ were statistically no different to those to ${positive}_{first}$ (5 vs. 5.5, $p=0.122$); Player 1 transfers to ${negative}_{last}$ were statistically no different to those to ${positive}_{last}$ (4.57 vs. 5.23, $p=0.431$); Player 2 transfers to ${negative}_{first}$ were statistically no different to those to ${positive}_{first}$ (28.78% vs. 29.78%, $p=0.783$); and Player 2 transfers to ${negative}_{last}$ were statistically no different to those to ${positive}_{last}$ (27.98% vs. 29.95%, $p=0.563$).

Table S3: Statistical Tests between positive and negative relationships by treatment

|  |  | **(1) First Impression** | | **(2) Last Interaction** | |
| --- | --- | --- | --- | --- | --- |
|  |  | **Z stat** | **p-value** | **Z stat** | **p-value** |
| **Market PE** | **Player 1 Transfers** | -1.682 | 0.093 | -2.396 | 0.017 |
|  | **Player 2 Transfers** | -1.422 | 0.155 | -2.387 | 0.017 |
| **Market IE** | **Player 1 Transfers** | -1.549 | 0.122 | -0.788 | 0.431 |
|  | **Player 2 Transfers** | -0.275 | 0.783 | -0.579 | 0.563 |

Table presents results from two-sided Mann-Whitney tests. Column (1) compares trust game transfers to ${positive}_{first}$ and ${negative}_{first}$ in Markets PE and IE. Column (2) compares trust game transfers to ${positive}_{last}$ and ${negative}_{last}$ in Markets PE and IE.

**Result 3 *(Betrayal aversion)***. Player 1 transfers to negative relationships in Market PE are smaller than transfers to negative relationships in Market IE.

***Support for Result 3****.* We continue to observe betrayal aversion in our experiment. Table S4 presents results from pairwise comparisons of Player 1 transfers to negative relationships defined by first impression and last interaction across treatments. Player 1 transfers to negative relationships in Market PE were significantly smaller than Player 1 transfers to negative relationships in Market IE when we define relationships by first impressions (3.53 vs. 5, $p=0.063$) and when we define relationships by last interactions (3.56 vs. 4.57, $p=0.026$).

Table S4: Comparison of trust game transfers to negative relationships across treatment

|  |  | **(1) Market PE vs. Market IE** | |
| --- | --- | --- | --- |
|  |  | **Z stat** | **p-value** |
| **Player 1 Transfers** | **First Impression** | -1.862 | 0.063 |
|  | **Last Interaction** | -2.22 | 0.026 |

Table presents results from a two-sided Mann-Whitney test and compares Player 1 transfers to negative relationships between Market PE and Market IE.

## **Comparisons across sessions**

One concern might be that our results are overly sensitive to differences in subject behavior between sessions, i.e. that sessions conducted at different times might have different internal dynamics that are driving our results. In this section, we evaluate whether subject behavior significantly varied across sessions in each treatment and analyze our trust game data using relationship types defined by subjects’ overall market experiences with their associates. Tables S5, S6 and S7 respectively present summary statistics on Player 1 and 2 transfers to negative relationships, positive relationships and subject pairs with no trading relationships across sessions by treatment. Unless otherwise noted, we use the Kruskal-Wallis test.

In Market PE, while the Player 2 transfers to negative relationships across sessions were statistically no different ($p=0.338$), at least one of the median Player 1 transfers to negative relationships by session was statistically different from the others ($p=0.032$). Statistical analysis revealed the converse results for Market IE; Player 1 transfers to negative relationships across sessions were statistically no different from the others ($p=0.199$) but at least one of the median Player 2 transfers to negative relationships by session was statistically different from the others ($p=0.007$).

Statistically, there was no meaningful difference between Player 1 transfers to positive relationships ($p= 0.336$) and between Player 2 transfers to positive relationships ($p=0.370$) across the 8 sessions in Market PE. Likewise, there was no significant difference between Player 1 transfers to positive relationship ($p=0.472$) and between Player 2 transfers to positive relationships ($p=0.436$) in Market IE.

Finally, there was no statistical difference between Player 1 transfers to Player 2s with whom they had no relationships across sessions in Market PE ($p=0.246$) and in Market IE ($p=0.242$). However, while there was no statistical difference between Player 2 transfers to Player 1s with whom they had no relationships across sessions in Market PE ($p=0.833$), at least one of the median Player 2 transfers to Player 1s with whom they had no relationships across sessions was statistically different from the others in Market IE ($p=0.069$).

Overall, although there is some variation, the results of the session comparisons above suggests that we need not be concerned that session level dynamics are driving our results, especially given that transfers to positive relationships is systematically the same.

Table S5: Summary of trust game transfers to negative relationships across sessions by treatment

|  |  | **Session** | | | | | | | |
| --- | --- | --- | --- | --- | --- | --- | --- | --- | --- |
|  |  | **1** | **2** | **3** | **4** | **5** | **6** | **7** | **8** |
| **Market PE** | **Player 1 Transfers** | 2.16  (0.14)  $n=19$ | 5.00  (-)  $n=1$ | 4.50  (0.25)  $n=10$ | 4.00  (0.18  $n=13$ | 3.60  (0.39)  $n=10$ | 2.09  (0.15)  $n=11$ | 5.17  (0.23)  $n=12$ | 3.50  (0.23)  $n=8$ |
|  | **Player 2 Transfers** | 23.95  (1.48  n=19 | 66.67  (-)  $n=1$ | 35.15  (1.86)  $n=11$ | 30.04  (2.40)  $n=13$ | 20.19  (2.55)  $n=10$ | 34.85  (3.42)  $n=11$ | 22.15  (1.57)  $n=12$ | 43.06  (2.83)  $n=8$ |
| **Market IE** | **Player 1 Transfers** | 5.00  (0.27)  $n=9$ | 4.50  (0.25)  $n=10$ | 6.36  (0.31)  $n=11$ | 5.08  (0.15)  $n=13$ | 4.38  (0.15)  $n=16$ | 4.36  (0.35)  $n=11$ | 2.57  (0.24)  $n=14$ | 4.86  (0.27)  $n=14$ |
|  | **Player 2 Transfers** | 9.33  (1.037)  $n=9$ | 57.63  (2.30)  $n=9$ | 32.96  (1.95)  $n=12$ | 23.91  (1.22)  $n=14$ | 18.54  (1.00)  $n=16$ | 24.05  (1.68)  $n=11$ | 42.76  (2.97)  $n=14$ | 18.21  (.21)  $n=14$ |

Standard errors are reported in parentheses. Percentage returned by Player 2 is calculated as Player 2’s token transfer divided by the respective Player 1’s tripled token transfer. For clarification, a dash indicates no mean or standard error is available to report.

Table S6: Summary of trust game transfers to positive relationships across sessions by treatment

|  |  | **Session** | | | | | | | |
| --- | --- | --- | --- | --- | --- | --- | --- | --- | --- |
|  |  | **1** | **2** | **3** | **4** | **5** | **6** | **7** | **8** |
| **Market PE** | **Player 1 Transfers** | 3.00  (0.71)  $n=2$ | 5.29  (0.59)  $n=7$ | 5.50  (1.77)  $n=2$ | 1.00  (0.71)  $n=2$ | 6.75  (0.55)  $n=4$ | 4.20  (0.17)  $n=5$ | 9.00  (-)  $n=1$ | 7.00  (0.85)  $n=5$ |
|  | **Player 2 Transfers** | 50.00  (-)  $n=2$ | 38.75  (4.07)  $n=8$ | 38.89  (12.90)  $n=3$ | 8.33  (5.89)  $n=2$ | 48.84  (3.26)  $n=4$ | 24.89  (3.57)  $n=5$ | 58.52  (8.38)  $n=2$ | 43.33  (4.48)  $n=6$ |
| **Market IE** | **Player 1 Transfers** | 6.00  (0.71)  $n=2$ | 8.00  (1.41)  $n=2$ | 6.00  (-)  $n=1$ | 4.33  (0.38)  $n=3$ | -  (-)  $n=0$ | 4.00  (2.12)  $n=2$ | -  (-)  $n=0$ | -  (-)  $n=0$ |
|  | **Player 2 Transfers** | 23.81  (16.84)  $n=2$ | 49.07  (2.33)  $n=3$ | -  (-)  $n=0$ | 0.00  (-)  $n=1$ | -  (-)  $n=0$ | 42.857  (-)  $n=1$ | -  (-)  $n=0$ | -  (-)  $n=0$ |

Standard errors are reported in parentheses. Percentage returned by Player 2 is calculated as Player 2’s token transfer divided by the respective Player 1’s tripled token transfer. For clarification, a dash indicates no mean or standard error is available to report.

Table S7: Summary of trust game transfers to no relationships across sessions by treatment

|  |  | **Session** | | | | | | | |
| --- | --- | --- | --- | --- | --- | --- | --- | --- | --- |
|  |  | **1** | **2** | **3** | **4** | **5** | **6** | **7** | **8** |
| **Market PE** | **Player 1 Transfers** | 2.53  (0.21)  $n=15$ | 5.50  (0.49)  $n=8$ | 6.75  (0.38)  $n=4$ | 3.00  (-)  $n=1$ | 4.50  (3.18)  $n=2$ | -  (-)  $n=0$ | 6.33  (0.77)  $n=3$ | 5.00  (1.53)  $n=3$ |
|  | **Player 2 Transfers** | 29.26  (1.47)  $n=15$ | 25.40  (3.83)  $n=7$ | 16.11  (7.46)  $n=2$ | 33.33  (-)  $n=1$ | 24.07  (17.02)  n=2 | -  (-)  $n=0$ | 23.33  (16.50)  $n=2$ | 66.66  (23.57)  $n=2$ |
| **Market IE** | **Player 1 Transfers** | 6.20  (0.38)  $n=5$ | 6.75  (0.72)  $n=4$ | 4.75  (0.24)  $n=4$ | -  (-)  $n=0$ | -  (-)  $n=0$ | 3.67  (1.54)  $n=3$ | 1.00  (0.71)  $n=2$ | 5.00  (1.41)  $n=2$ |
|  | **Player 2 Transfers** | 6.33  (2.17)  $n=5$ | 53.97  (3.02)  $n=4$ | 41.25  (2.95)  $n=4$ | 40.00  (-)  $n=1$ | -  (-)  $n=0$ | 17.59  (4.56)  $n=4$ | 25.00  (17.68)  $n=2$ | 30.16  (17.96)  $n=2$ |

Standard errors are reported in parentheses. Percentage returned by Player 2 is calculated as Player 2’s token transfer divided by the respective Player 1’s tripled token transfer. For clarification, a dash indicates no mean or standard error is available to report.

## **Further suggestive evidence of the effect of personal exchanges on trust and reciprocity**

We have further suggestive experimental evidence that, unlike those in the market where exchanges were more impersonal, subjects in the market where exchanges were more personal genuinely learnt whom to trust. For another study (not reported here), we implemented a version of our Markets PE and IE treatments where every aspect of the design is identical to Markets PE and IE treatments except for the first game. In this new treatment, subjects performed a slider task developed by Gill and Prowse (2018) – not a market game – followed by the trust game. The slider task serves as a useful parallel to our market treatments, as it does not involve market exchanges. Furthermore, since subjects performed the slider task in isolation from other subjects, the Player 1 and Player 2 transfers in the slider treatment may be interpreted as the baseline levels of trust and reciprocity the subject pool at George Mason University possess. Statistical analysis revealed that: Player 1 transfers in the slider treatment are significantly larger than Player 1 transfers in Market PE (5.32 vs. 4.02, $p=0.002$) but statistically no different to those in Market IE (5.32 vs. 4.71, $p=0.192$); and Player 2 transfers in the slider treatment are statistically no different to those in Market PE (30.16% vs. 31.38%, $p=0.860$) and Market IE (30.16% vs. 28.27%, $p=0.462$). If it is indeed reasonable to interpret trust game transfers in the slider treatment as subject pool baselines, this is an additional (suggestive) evidence that hints at how the market where exchanges are more personal teach people whom to trust but the market where exchanges are more impersonal do not.
